# Synthesis and Evaluation of AS1411-Lenalidomide-Targeted Degradation Chimera in Antitumor Therapy

**DOI:** 10.3390/ph18121867

**Published:** 2025-12-07

**Authors:** Xueling Ma, Shuangshuang Liu, Xiao Dong, Xiuhua Li, Feiyan Wang, Jiawei Zhang, Zhenfang Xu, Weiguo Shi, Aiping Zheng, Aiping Zhang, Xuesong Feng, Liang Xu

**Affiliations:** 1 School of Public Health, China Medical University, Shenyang 110122, China; 2 Academy of Military Medical Sciences, Beijing 100850, China; 3 School of Pharmacy, China Medical University, Shenyang 110122, China; 4 College of Pharmacy, Shanxi Medical University, Taiyuan 030000, China; 5 Department of Anesthesiology, The First Affiliated Hospital of Jinan University, Guangzhou 510642, China

**Keywords:** aptamer–PROTAC conjugates, nucleolin, targeted protein degradation, AS1411 aptamer, lenalidomide

## Abstract

**Background**: High expression of nucleolin (NCL) on the surface of tumor cells is closely associated with disease progression and poor prognosis. The aptamer–PROTAC conjugate (APC) technology provides a novel molecular design strategy for the targeted degradation of NCL. **Methods**: Based on the principles of PROTAC technology and chemical modification techniques, in this study, a series of AS1411-lenalidomide chimeras featuring different linker structures were designed and synthesized for the specific purpose of targeted degradation of NCL. Four AS1411-PROTACs (C1–C4) were successfully constructed via a click chemistry strategy, and their structures were validated. **Results**: In vitro experimental results showed that C4 exhibited the most optimal activity, significantly downregulating NCL expression and inhibiting the proliferation of breast cancer cells (MCF-7). Notably, the activity of C4 remained unaltered regardless of the annealing process. Mechanistic studies demonstrated that C4 induced NCL degradation through the ubiquitin–proteasome pathway while also promoting apoptosis and cell cycle arrest. In a nude mouse tumor model, C4 displayed potent antitumor efficacy, with no discernible signs of obvious systemic toxicity. **Conclusions**: This study provides compelling evidence demonstrating that C4 is a highly promising anticancer compound. It also provides important evidence for the development of novel nucleic acid aptamer–PROTAC conjugate drugs for more clinical applications.

## 1. Introduction

Nucleolin (NCL), a multifunctional RNA-binding protein, exhibits significantly higher expression on the surface of various tumor cells compared to normal cells. This differential distribution makes it an ideal target for tumor-specific targeted therapy in recent years [1,2]. NCL not only participates in the regulation of tumor cell proliferation, migration, and apoptosis but also influences the activation of multiple oncogenic signaling pathways through its nucleocytoplasmic shuttling mechanism. Notably, aberrant expression of NCL is closely associated with disease progression and poor prognosis in malignancies such as breast cancer, glioma, renal cell carcinoma, and leukemia [3].

In the field of targeted protein degradation (TPD), proteolysis-targeting chimeras (PROTACs) have garnered significant attention due to their ability to achieve catalytic degradation of target proteins via the ubiquitin–proteasome system (UPS) [1,4]. PROTAC is a heterobifunctional molecule. One end of the molecule connects to the ligand of the target protein, and the other end connects to the ligand of the E3 ligase, with the two parts joined by a linker. PROTAC binds to the target protein and E3 ligase simultaneously, forming a ternary complex that allows for spatial proximity of the target protein to the E3 ligase. This facilitates the ubiquitination of the target protein, marking it for recognition and degradation by the proteasome [5]. Conventional PROTAC molecules, typically composed of small-molecule ligands, face limitations such as poor solubility and restricted cellular selectivity [6].

Nucleic acid aptamers possess high affinity and specificity comparable to antibodies, along with advantages like low immunogenicity, ease of chemical modification, and superior tissue penetration [7,8,9]. Aptamer–PROTAC conjugate (APC) technology represents a cutting-edge research direction in recent years, offering advantages such as improved molecular water solubility, enhanced serum stability, and strengthened binding affinity toward “undruggable” proteins [10,11,12]. Despite these notable advantages, among the limited number of reported studies, the majority have focused on antitumor applications, and the structural diversity of molecules remains constrained by factors including linker types, conjugation modes, and the selection of E3 ligase ligands [13,14]. Additionally, the literature has indicated that the frequently used NCL-targeting aptamer AS1411 exhibits high structural polymorphism, adopting multiple conformations under different environmental conditions; however, the extent to which this conformational variation interferes with its biological activity remains poorly characterized [15,16].

In this study, we designed and synthesized a series of novel AS1411-lenalidomide chimeras with targeted degradation mechanisms based on PROTAC technology and chemical modification strategies by designing linkers of different lengths and structures. These chimeras feature three critical functional domains: (1) AS1411 aptamer as the NCL-targeting module, (2) lenalidomide derivatives as ligands recruiting the Cereblon (CRBN) E3 ubiquitin ligase, and (3) regulating ligation arms and atom selection. This work expands the molecular diversity of aptamer–PROTAC and provides a new direction for the development of novel antitumor drugs.

## 2. Results

### 2.1. Design and Synthesis of AS1411-PROTACs

**Scheme 1:**
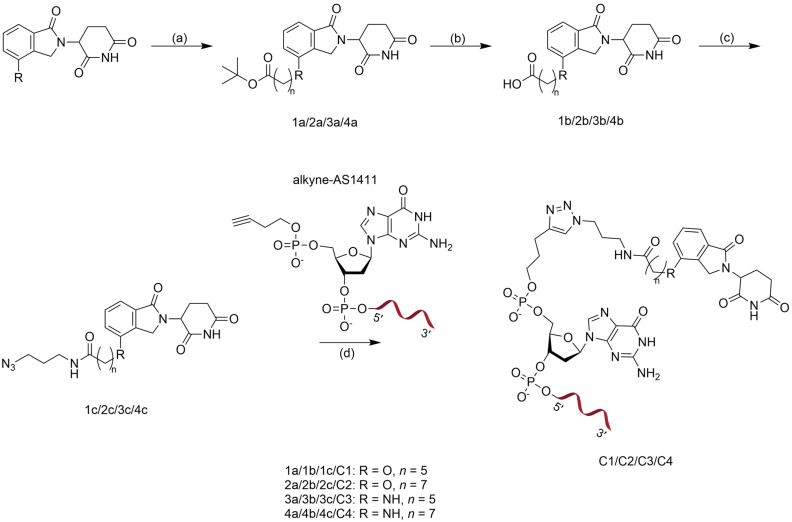
Synthetic roadmap of AS1411-PROTACs. Reagents and conditions: (a) *tert*-butyl 6-bromohexanoate/*tert*-butyl 8-bromooctanoate, DIPEA, NMP, 120 °C, 15 h; (b) TFA, DCM, rt, 2 h; (c) HBTU, HOBt, 3-azido-1-propylamine, TFA, DMF, rt, 12 h; (d) TEAA, DMSO, Cu (II)-TBTA complex, sodium ascorbate, 37 °C.

We explored a range of synthesis strategies (Scheme 1 and Schemes S1–S4, Table S1), and ultimately chose to introduce an alkynyl moiety at the 5′ end of the AS1411 and incorporate an azide group into the lenalidomide derivatives. Under the conditions of Cu(I) catalytic [17], a click chemical cycloaddition reaction between the alkynyl moiety and the azide group can yield 1,4-disubstituted-1,2,3-triazole. Based on this strategy, four lenalidomide derivatives (1c, 2c, 3c, and 4c) with terminal azide groups were successfully synthesized and conjugated with the 5′-terminal alkynyl-modified AS1411 (alkyne-AS1411, which was custom-synthesized by Beijing Qinke Biotechnology Co., Ltd.), resulting in four AS1411-PROTACs (C1–C4). The structures were confirmed by MOLDI-TOF MS (Table 1, Figure S1). Taking C4 as an example, according to HPLC analysis, the peak exit positions of C4 and the alkyne-AS1411 were different (Figure S2a). Absorption peaks at 225 nm and 260 nm also showed notable shifts between C4 and Alkyne-AS1411 (Figure S2b). The gel electrophoresis results showed that the positions of the electrophoresis bands of AS1411 and C1–C4 were different (Figure S2c). These results illustrated the successful conjugation of lenalidomide.

### 2.2. C4 Exhibits Definite Antitumor Efficacy In Vitro

**Figure 1:**
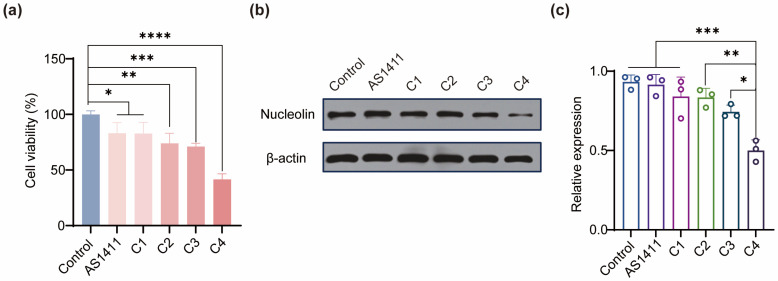
Active primary screening of AS1411-PROTACs: (**a**) Proliferation inhibition of MCF-7 cells treated with 1 μM aptamers for 12 h. (**b**) The NCL levels in MCF-7 cells were evaluated by Western blot after treating with 1 μM aptamers for 12 h. (**c**) Statistical analysis of the expression of NCL in MCF-7 cells. The colored circles (blue, purple, pink, green, cyan, black) correspond to the biological replicates of each experimental group (Control, AS1411, C1, C2, C3, C4). ns: no significant difference. * *p* < 0.05, ** *p* < 0.01, *** *p* < 0.001, **** *p* < 0.0001.

AS1411 has been demonstrated to bind to NCL and inhibit the growth of malignant breast cancer cells without affecting cytoplasmic NCL expression levels [18,19]. The cell proliferation inhibition test was used to screen for chimeric compounds. CCK-8 assay results revealed that treatment with AS1411, C1, C2, C3, or C4 led to varying degrees of reduction in MCF-7 cell viability, among which the decrease in cell viability was more significant after treatment with C4 (Figure 1a). Moreover, C4 exhibited a clear dose-dependent relationship, with a half-maximal inhibitory concentration (IC_50_) of 0.9 μM in MCF-7 cells (Figure S3).

At the protein expression level, Western blot analysis confirmed that C4 treatment resulted in lower NCL expression compared to other groups (Figure 1b,c), suggesting that the superior anti-proliferative effects of C4 might be ascribed to more efficient NCL downregulation. Based on these results, C4 was chosen for further evaluation in cellular and animal models.

### 2.3. The Advanced Structure and Activity of C4

**Figure 2:**
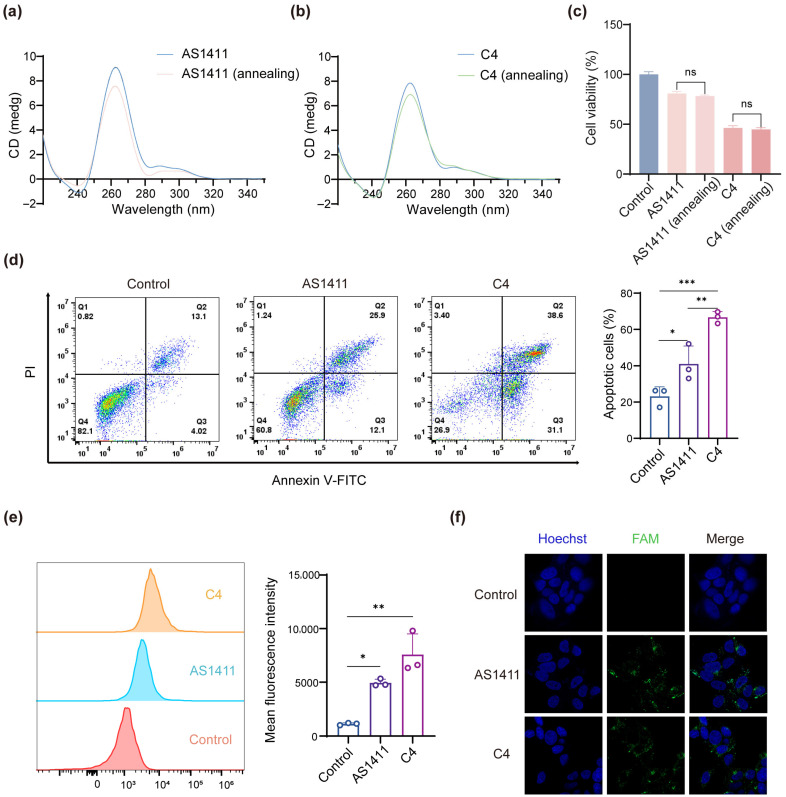
The advanced structure and activity of C4: (**a**,**b**) Circular dichroism of annealed and not-annealed AS1411 and C4. (**c**) Antitumor activity of annealed and non-annealed AS1411 and C4 in vitro. (**d**) The cell apoptosis of MCF-7 cells treated with AS1411 and C4 detected by flow cytometry. (**e**) Representative fluorescence histograms of the AS1411 and C4 uptake pathway in MCF-7 cells. In (**d**,**e**), the colored circles (blue, purple, pink) correspond to the biological replicates of each experimental group (Control, AS1411, C4). (**f**) A confocal laser scanning microscope was used to observe the fluorescence of AS1411 and C4 uptake by MCF-7 cells. “Hoechst” (blue) labels cell nuclei, and “FAM” (green) labels AS1411/C4. ns: no significant difference. * *p* < 0.05, ** *p* < 0.01, *** *p* < 0.001.

AS1411 exerts its antitumor activity by forming a G-quadruplex structure that binds to NCL [20]. CD analysis revealed that both AS1411 and C4 exhibit similar spectral signatures, with a positive peak at 265 nm and a negative peak at 240 nm, typical of a parallel G-quadruplex spectrum [15,16] (Figure 2a,b). This indicated that the introduction of the linker and lenalidomide in the C4 chimera did not disrupt the formation of advanced structures in the AS1411 domain. Neither AS1411 nor C4 showed significant differences in cell viability inhibition compared to their annealed counterparts (Figure 2c), suggesting that conformational diversity induced by annealing did not affect biological activity. In addition, the thermal stability of the nucleic acid was evaluated by measuring the change in absorbance with temperature. Thermal denaturation curves demonstrated that C4 has a higher melting temperature (Tm = 66.62 °C) than AS1411 (Tm = 62.75 °C) (Figure S4). This enhanced stability likely arises from altered base stacking and hydrogen bonding interactions due to the incorporation of lenalidomide and the linker.

The scratch wound assay demonstrated that C4 treatment significantly suppressed MCF-7 cell migration compared to the control and AS1411 groups (Figure S5), which may be related to its induced NCL degradation. Flow cytometry analysis revealed that both AS1411 and C4 promoted apoptosis in MCF-7 cells, with C4 exhibiting stronger pro-apoptotic effects than AS1411 (Figure 2d). Further, C4 treatment reduced the G0/G1 population while increasing G2/M phase cells, indicating that C4, like AS1411, led to cell cycle arrest in G2/M phase and had a more significant effect (Figure S6).

Previous studies have demonstrated that AS1411 can specifically bind to breast cancer cells by recognizing cell surface NCL [21,22]. To assess whether C4 retains this binding specificity, cells were incubated with FAM-labeled AS1411 and C4. Based on initial condition exploration, a 10 μM concentration was used in cell apoptosis, cell cycle, and cellular uptake assays to meet the sensitivity requirements of these detection indicators. As shown in Figure 2e, both AS1411 and C4 groups exhibited significant fluorescence peak shifts in MCF-7 cells, with the C4 group showing a higher mean fluorescence intensity (though not statistically significant), indicating that the small-molecule ligand in C4 did not interfere with its binding specificity to breast cancer cells. Additionally, the selective binding ability of C4 was further confirmed by CLSM (Figure 2f). To further verify the broad selectivity of C4, as shown in Figures S7 and S8, C4 was efficiently internalized in HeLa cells, whereas no uptake of C4 was observed in BEAS-2B and Vero cells. These results demonstrated that C4 maintains excellent binding affinity for cancer cells.

### 2.4. C4 Induces the Degradation of NCL In Vitro

**Figure 3:**
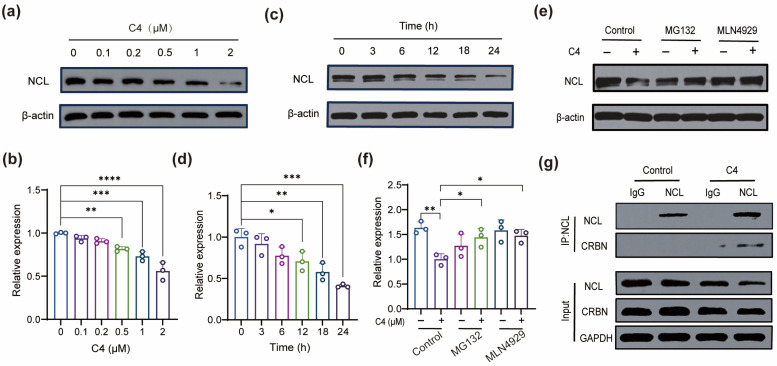
Degradation of NCL induced by C4: (**a**) Level of NCL in MCF-7 cells after treatment with C4 at the indicated concentrations for 12 h. (**b**) Statistical analysis of the expression of NCL in (**a**). The colored circles (blue, purple, pink, green, cyan, black) correspond to the biological replicates of each C4 concentration group. (**c**) Level of NCL in MCF-7 cells after treatment with 1 μM C4 at the indicated time points. (**d**) Statistical analysis of the expression of NCL in (**c**). The colored circles (blue, purple, pink, green, cyan, black) correspond to the biological replicates of each time point group. (**e**) Level of NCL in MCF-7 cells after treatment with 1 μM C4 for 12 h, in the presence or absence of 10 μM proteasome inhibitor MG132 or NEDD8-activating enzyme inhibitor MLN4924. (**f**) Statistical analysis of the expression of NCL in (**e**). The colored circles (blue, purple, pink, green, cyan, black) correspond to the biological replicates of each treatment group (Control, Control + C4, MG132, MG132 + C4, MLN4929, MLN4929 + C4). (**g**) MCF-7 cells were treated with C4 or AS1411 as a control for 12 h, followed by incubation with IgG or anti-NCL antibodies and co-immunoprecipitation for CRBN-NCL interaction determination. * *p* < 0.05, ** *p* < 0.01, *** *p* < 0.001, **** *p* < 0.0001.

The regulatory effects of C4 on NCL expression and its underlying degradation mechanism were thoroughly characterized using Western blot analysis. As depicted in Figure 3a,b, NCL levels in MCF-7 cells decreased with increasing concentrations of C4, indicating that the protein-degrading activity of C4 is dose-dependent. As shown in Figure 3c,d, prolonged exposure to C4 led to a gradual reduction in NCL levels, demonstrating a time-dependent effect. To verify whether the C4-mediated degradation of NCL depends on the ubiquitin–proteasome pathway, we added the proteasome inhibitor MG132 or the NEDD8-activating enzyme inhibitor MLN4929 prior to C4 treatment. Quantitative analysis of Western blot results showed that after treatment with MG132 or MLN4929, the relative expression level of NCL significantly increased compared with the C4-only treatment group (*p* < 0.05), and the elevated level was close to that of the control group (Figure 3e,f). These findings suggested that C4 induced NCL degradation through the ubiquitin-proteasome pathway, thereby suppressing the proliferation of breast cancer cells. Furthermore, ternary complex formation is a prerequisite for PROTAC-mediated protein degradation. We thus analyzed whether C4 could promote the formation of NCL−C4−CRBN ternary complexes. Co-immunoprecipitation revealed a significant enhancement in the affinity between NCL and CRBN in the presence of C4 but not control AS1411 (Figure 3g).

### 2.5. C4 Exhibits High Antitumor Efficacy In Vivo

**Figure 4:**
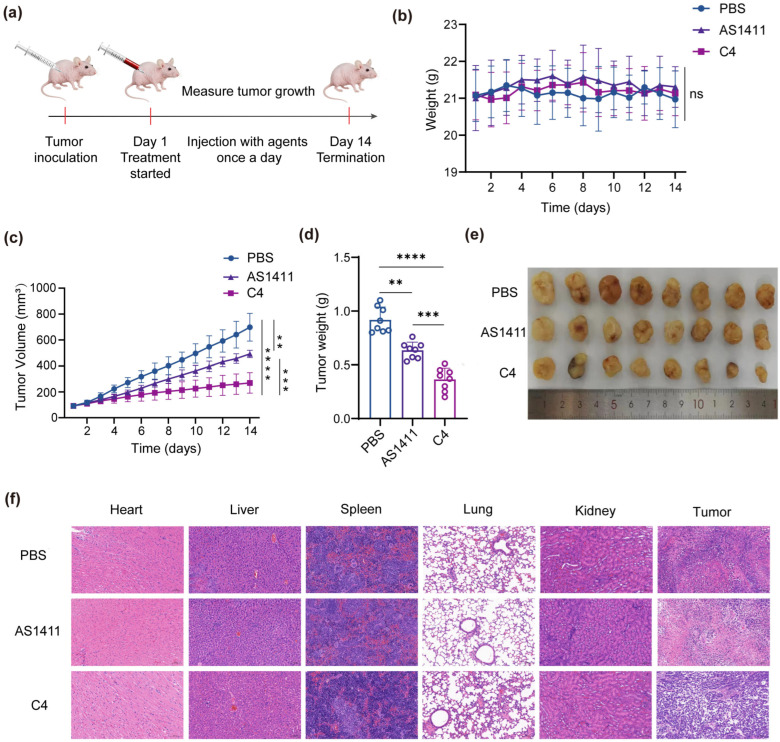
Antitumor activity of C4, AS1411, and PBS in vivo: (**a**) Schematic diagram of the timeline of in vivo animal studies. (**b**) Bodyweight curves of MCF-7 tumor-bearing mice after different treatments. (**c**) Growth curves of MCF-7 tumor xenografts in mice after different treatments. (**d**) Tumor weights of the tumor-bearing mice after different treatments. The colored circles (blue, purple, pink) correspond to the biological replicates of each experimental group (PBS, AS1411, C4). (**e**) Images of tumor tissues isolated from mice after different treatments. (**f**) Representative images of H&E staining of heart, liver, spleen, lung, kidney, stomach, and tumor tissues. Scale bar: 100 μm. The data are presented as the mean ± SD, *n* = 8. ns: no significant difference. ** *p* < 0.01, *** *p* < 0.001, **** *p* < 0.0001.

The antitumor efficacy of C4 was further evaluated in MCF-7 tumor-bearing mice. The treatment regimen consisted of intratumoral injections once daily for 14 days (Figure 4a). Throughout the experiment, the mice in all treatment groups displayed excellent tolerance to the intervention, with no significant reduction in body weight (Figure 4b), indicating a favorable safety profile. After the treatment, tumor specimens were excised, photographed, and weighed from the mice, with the average tumor weight calculated for each group. As illustrated in Figure 4c–e, tumor growth was notably suppressed in the C4 treatment group compared to the control and AS1411 groups. For histological analysis, major organs (heart, liver, spleen, lungs, and kidneys) and tumors were excised from the mice. H&E staining of tumor sections from the C4 group revealed prominent nuclear condensation and fragmentation, accompanied by extensive necrotic regions, which further validated the potent antitumor activity of C4. In contrast, no obvious tissue damage or morphological abnormalities were detected in the major organs of C4-treated mice (Figure 4f). These results collectively indicated that C4 is a promising anticancer compound.

## 3. Discussion

In contrast to the classic PROTAC technology based on small-molecule ligands, aptamer–PROTAC technology enables the targeted degradation of NCL, offering a novel molecular category and perspective for cancer therapy. Currently, optimizing molecular architectures and expanding structural diversity stand as the cutting-edge research directions in this technical domain.

In an effort to broaden structural diversity and explore novel linker structures and bonding modes, we initially conducted investigations on a range of synthetic strategies, including azide-alkyne-based, halogen-thiol-based, halogenated acetyl bromide-thiol-based, bromobenzene-thiol-based, and N-hydroxy-succinimidyl ester-amine-based strategies (Scheme 1 and Schemes S1–S4, Table S1). Unfortunately, only the azide-alkyne-based strategy successfully yielded the target chimeric molecules. The other strategies were abandoned due to either failure to detect the target molecules or excessive side reactions that complicated purification at different stages. Compared with pomalidomide, which has a similar structure but more diverse derivatives, the relatively high reactivity of the -CH_2_- group on the isoindole ring of lenalidomide may be a key reason for the failure of other synthetic routes. Eventually, four AS1411-PROTACs (C1, C2, C3, and C4) were successfully synthesized using the click chemistry strategy between alkynyl and azido groups. Their structures were confirmed by various characterization methods. This synthetic strategy ameliorates the drawbacks of numerous side reactions and difficult purification in the synthesis of lenalidomide derivatives [23], expands the synthetic pathways for aptamer–PROTAC, and features rapid reaction efficiency with high yields, which is conducive to large-scale applications in the future.

In both the cell viability assay and Western blot analysis, C4 exhibited stronger cellular inhibitory activity and induced lower protein levels when compared to the other groups. This superior performance was presumably associated with the length and chemical properties of the linker within its structure, which influenced the spatial proximity and binding efficiency with both NCL and the CRBN E3 ligase. Furthermore, research has indicated that AS1411 can form at least eight distinct G-quadruplex conformations under slow annealing conditions by modifying the cooling rate during the annealing step [24]. Consistently, we observed that the annealed samples of both AS1411 and C4 displayed weaker positive absorption peaks at 260 nm compared to samples prepared by directly dissolving the solid samples (Figure 2a,b). This phenomenon might be attributed to the structural dynamics induced by the annealing process [25]. However, cellular experiments demonstrated that the annealing treatment did not significantly affect the results (Figure 2c), suggesting that the conformational diversity generated by annealing did not impact cell viability. This implied that AS1411 and C4 may undergo favorable conformational dynamic transitions that enhance their interaction with NCL. Meanwhile, compared with AS1411, the enhanced thermal stability of C4 (Tm = 66.62 °C) not only serves as a direct hallmark of successful structural modification but also provides a robust foundation for optimizing its binding characteristics with NCL at the target-binding kinetic level. Dailey et al. [24] reported that the conformational polymorphism of AS1411 results in fluctuations in its binding specificity to NCL. Following thermal stability enhancement via flanking duplex modification, AS1411 exhibited reduced conformational disorder, which was accompanied by a concurrent improvement in its binding stability to NCL. This observation is highly consistent with the findings of Alkhamis et al. [26], who proposed a positive correlation between the thermal stability of aptamers and their target-binding specificity. Specifically, enhancing thermal stability through structural modification can lock the functional conformation of aptamers, thereby potentiating their recognition specificity toward target molecules. Therefore, it is hypothesized that the increased thermal stability of C4 will not significantly alter its NCL-binding kinetic parameters; instead, it can optimize the binding specificity to NCL by mitigating nonspecific interactions arising from conformational disorder. These findings not only validated the feasibility of the aptamer–PROTAC strategy but also highlighted the critical role of linker design in regulating protein degradation efficiency.

Previous studies have demonstrated that AS1411 can specifically bind to breast cancer cells by recognizing NCL on the cell surface. CD assay confirmed that the incorporation of the small-molecule ligand into C4 did not disrupt the G-quadruplex structure of its AS1411 domain, which is fundamental to maintaining its targeting capability. Cell uptake experiments using flow cytometry and CLSM showed that C4 was efficiently internalized by MCF-7 tumor cells but not by normal BEAS-2B cells, exhibiting excellent cell-type selectivity. This selectivity was further validated in HeLa tumor cells and Vero normal cells, where C4 maintained high uptake efficiency in cancer cells and low nonspecific internalization in normal cells, confirming its stable targeting ability across different tumor and normal cell lines.

Compared with the control and AS1411 groups, the C4 group significantly inhibited the migration of MCF-7 cells. Furthermore, numerous previous studies have demonstrated that treatment of tumor cells with AS1411 increases cell cycle arrest at the S and G2/M phases [27,28,29,30]. Consistent with the effects of AS1411, C4 treatment induced cell cycle arrest at the G2/M phase, and this effect was more pronounced (Figure S6). These results were intricately associated with the induction of NCL dysfunction. Further mechanistic studies confirmed that C4 degrades NCL via the ubiquitin–proteasome pathway with the formation of NCL−C4−CRBN ternary complexes, and this process is concentration- and time-dependent. In vivo experiments, C4 significantly suppressed the growth of xenografted tumors in nude mice without causing obvious organ toxicity. Notably, AS1411 inhibits tumor cell growth solely by binding to NCL, while classic PROTACs, although capable of degrading target proteins, tend to lack selectivity. C4 integrates the advantages of both approaches: it retains the tumor-targeting property of AS1411 and possesses the catalytic degradation function of PROTACs, which contributes to its superior performance.

Despite the encouraging results, this study has certain limitations. First, the application of cell lines and tumor models is singular, focusing only on breast cancer MCF-7 cells and nude mouse subcutaneous xenograft models, without covering other tumor types with high NCL expression (such as glioma and leukemia), models of different pathological subtypes or drug-resistant phenotypes, nor verifying with orthotopic and metastatic tumor models. This makes it difficult to reflect the heterogeneity of clinical tumors and limits the generalizability of the conclusions. Second, only intratumoral injection was used for drug administration in the in vivo experiments, and clinically common routes such as intravenous injection and oral administration were not explored. This makes it impossible to clarify the impact of different administration routes on drug absorption, distribution, and efficacy, which is not conducive to clinical translation. Third, there is a lack of systematic pharmacokinetic data (such as blood drug concentration, half-life, tissue distribution, etc.) and long-term pharmacodynamic tracking, and the effect of combined drug use has not been evaluated, making it difficult to optimize treatment regimens and comprehensively judge clinical value. Conducting detailed pharmacokinetic and pharmacodynamic studies is crucial for optimizing the therapeutic index and minimizing potential side effects.

## 4. Materials and Methods

### 4.1. Chemical Synthesis and Structural Characterization of the Target Compounds

All commercial reagents and solvents were utilized without further treatment unless otherwise specified. Progress of chemical reactions was monitored by thin-layer chromatography (TLC) using Energy GF254 high-performance TLC silica gel plates (model: BM000010; dimensions: 2.5 × 7.5 cm; coating thickness: 0.2–0.25 mm, bound with sodium carboxymethyl cellulose), supplied by Anhui Zekai Technology Co., Ltd. (Hefei, China). Visualization of the developed plates was achieved under ultraviolet (UV) light (254 nm). Purification of reaction products was performed via column chromatography using STEEMA column chromatography silica gel (200–300 mesh), supplied by Beijing Jianqiang Weiye Technology Co., Ltd. (Beijing, China). The nucleic acid sequences, including 5′-terminal alkynyl-modified AS1411 (alkyne-AS1411), were custom-synthesized by Beijing Qinke Biotechnology Co., Ltd. (Beijing, China). Specifically, the terminal alkynyl group was introduced at the 5′-end of the AS1411 sequence using alkynyl phosphoramidite monomer during the solid-phase synthesis process. The sequences were identified using matrix-assisted laser desorption/ionization time-of-flight mass spectrometry (MALDI-TOF MS) (KRATOS analysis, Shimadzu Group, Kyoto, Japan). The synthesized nucleic acid sequences were monitored by SPD-10Avp High-Performance Liquid Chromatograph (Shimadzu, Kyoto, Japan). The synthesized nucleic acid sequences were dissolved in a small amount of deionized water, and their absorbance at 260 nm was measured using a Cary100 UV–vis spectrophotometer (Varian, Palo Alto, CA, USA) to determine the concentration (OD/μL). According to the desired concentration of the nucleic acid solution, the necessary OD value can be calculated using the following formulas: ε = 15.2 × A + 12.01 × G + 8.4 × T + 7.05 × C and OD = ε × c × 10^−3^ × V, where ε is the molar absorptivity coefficient. Synthesis of compounds can be found in the Supplementary Materials.

### 4.2. Spectroscopic Characterization

^1^H NMR and ^13^C NMR spectra were acquired on a BRUKER AVANCE NEO 600 MHz Superconducting Nuclear Magnetic Resonance Spectrometer (Bruker, Fällanden, Switzerland). The operating frequencies were 600 MHz (for ^1^H NMR) and 150 MHz (for ^13^C NMR). Samples were dissolved in DMSO-d_6_, with residual solvent peaks used as internal references. Chemical shifts (δ) are reported in parts per million (ppm), coupling constants (J) in hertz (Hz), and peak multiplicities are abbreviated as: s (singlet), d (doublet), t (triplet), q (quartet), m (multiplet), br (broad).

Electrospray ionization mass spectrometry (ESI-MS) and high-resolution mass spectrometry (HRMS) data were acquired using an Agilent 1260/G6230A Liquid Chromatography–Mass Spectrometry (LC-MS) System (Agilent Technologies, Santa Clara, CA, USA). The ionization mode was set to positive electrospray ionization (ESI+), and the mass scan range was *m*/*z* 50–1000.

### 4.3. UV–Vis Spectroscopy Analysis

The samples (0.5 OD) were dissolved in 1ml of PBS buffer (containing 100 mmol/L KCl). The UV spectra were acquired on a Cary100 UV–vis spectrophotometer by using a scanning speed of 100 nm/min with the appropriate baseline subtracted in the range 200–350 nm.

### 4.4. Electrophoresis Analysis

Pre-run the prepared gel at 90 V in 1× TBE buffer for 30 min. Load the samples and run electrophoresis at a constant voltage of 90 V at room temperature. After 1.5 h, stop the electrophoresis and then place the gel on a silica plate and irradiate it with a handheld UV lamp at a wavelength of 254 nm. Take photos using a mobile phone and analyze the photos using ImageJ 1.54g software.

### 4.5. Cell Lines and Culture Methods

The MCF-7, BEAS-2B, HeLa, and Vero cells were purchased from Wuhan Servicebio Technology Co., Ltd. (Wuhan, China), China. All cells were cultured in DMEM medium containing 1% penicillin–streptomycin solution and 10% fetal bovine serum at 37 °C and 5% CO_2_. Cells were grown to 70–80% confluency in dishes or cell culture plates and treated under various conditions as indicated.

### 4.6. Cell Viability Assay

The cell proliferation assay was carried out using the CCK-8 kit in accordance with the manufacturer’s instructions. Briefly, MCF-7 cells were seeded in 96-well plates at 5 × 10^3^ cells/well and cultured in DMEM medium containing 10% FBS for 24 h. Subsequently, the cells were exposed to various concentrations of the compounds (1 μM) for a duration of 12 h. After treatment, the culture supernatant was completely removed, and 100 μL of CCK-8 solution was added to each well, followed by an additional incubation of 1 h at 37 °C. The optical density (OD 450) was measured using a microplate reader (E-max; Molecular Devices, Sunnyvale, CA, USA). Based on the data obtained, the cell survival curve was plotted, and the IC_50_ value was calculated.

### 4.7. Measurement of Thermal Transition Curves (Tms)

About 1 OD of sample was dissolved in 1 mL of PBS buffer (containing 100 mmol/L KCl), heated at 95 °C for 5 min, slowly cooled to 25 °C, and then the change in absorbance with temperature was measured at a wavelength of 295 nm using a Cary-100 Bio UV-visible spectrophotometer at a rate of 1 °C/min from 25 °C to 95 °C, and the change in absorbance with temperature was measured at a wavelength of 295 nm. The Tm value was calculated from the first derivative plots of absorbance vs. temperature.

### 4.8. Circular Dichroism (CD) Spectroscopy Analysis

CD spectra of the samples, dissolved in 200 μL of PBS buffer (containing 100 mmol/L KCl), were recorded on a spectropolarimeter (MOS-450; Bio-Logic, Inc., Seyssinet-Pariset, France). The temperature was 25 °C, and the scanning wavelength range was 220–350 nm.

### 4.9. Western Blot Analysis

The cells were washed with ice-cold PBS and lysed on ice with RIPA buffer. Protein concentration was determined using the BCA protein detection kit. Samples with equal protein concentrations were loaded onto a protein separation system, protein samples were separated by SDS-PAGE, and then the proteins were transferred to polyvinylidene fluoride (PVDF) membranes. The PVDF membranes were blocked with 5% milk at room temperature. After washing three times with TBST buffer, the membranes were incubated with diluted corresponding primary antibodies at 4 °C overnight. After washing with TBST buffer, the membranes were incubated with HRP-conjugated secondary antibodies on a shaker at room temperature. HRP antibodies were detected using the SuperSignal West Pico PLUS chemiluminescent substrate (Beijing Pulilai Gene Technology Co., Ltd., Beijing, China). The blots were imaged with a chemiluminescence imaging system (JDFS-3000P, Wuhan Servicebio Technology Co., Ltd., China).

### 4.10. Cell Scratch Wound Assay

MCF-7 cells were seeded in a 12-well plate to grow until creating 95% to 100% confluent monolayers. The monolayers were scratched with a sterile pipette tip and washed with serum-free medium. MCF-7 cells were treated with the samples (1 μM) in serum-free medium. Images from the same wounded fields were obtained at 0, 12, and 24 h under AOSVI Imaging. The wound-healing area (area not covered by cells) was calculated using ImageJ 1.54g software.

### 4.11. Cell Apoptosis Analysis

The annexin V–fluorescein isothiocyanate (FITC)/propidium iodide (PI) apoptosis detection kit was utilized. MCF-7 cells were seeded into 6-well plates and incubated with the samples (10 μM). After 48 h, cells were collected and suspended in binding buffer containing annexin V–fluorescein isothiocyanate (FITC) and propidium iodide (PI) for a 15 min period. Finally, the samples were subjected to flow cytometry (FACSCalibur, BD Biosciences, Franklin Lakes, NJ, USA).

### 4.12. Cell Cycle Assay

MCF-7 cells were seeded into 6-well plates and incubated with the samples (10 μM). After 48 h, cells were harvested and gently rinsed with PBS, followed by fixation in cold 70% ethanol at 20 °C overnight. The fixed cells were washed with PBS and then treated with PI/RNase Staining Buffer for 15 min in the dark at 37 °C. Finally, the samples were subjected to flow cytometry (BD Bioscience, Oxford, UK).

### 4.13. Cellular Uptake

The cellular uptake ability of C4 was studied using flow cytometry and confocal scanning laser microscopy (CLSM). Cells were seeded into 12-well culture plates without (for flow cytometry detection) or with (for CLSM observation) glass coverslips at a density of 1 × 10^5^ cells per well and allowed to attach and grow overnight. FAM-labeled AS1411 and C4 (10 μM) were added and incubated for 4 h. For flow cytometry, the cells were washed three times with 300 µL PBS, and then 300 µL 4% paraformaldehyde was added, followed immediately by flow cytometric analysis. For CLSM, the cells were washed three times with 1× PBS, and the cell nuclei were stained with Hoechst for 10 min. Subsequently, all samples were observed with CLSM (TSC SP8 STED 3X, Leica Inc., Jena, Germany).

### 4.14. Co-Immunoprecipitation (Co-IP) Assay

Briefly, MCF-7 cells after different treatments were lysed using IP lysis buffer supplemented with a proteinase inhibitor cocktail. After centrifugation at 13,000× *g* at 4 °C, the supernatant was incubated with the corresponding primary antibody overnight at 4 °C and then coupled to Protein A/G agarose beads at room temperature for 1 h. To reduce nonspecific binding, the beads were extensively washed five times using IP lysis buffer. The immunoprecipitated complexes were then resuspended in a loading buffer, heated at 95 °C for 5 min, and subsequently analyzed by Western blotting.

### 4.15. In Vivo Anticancer Studies

The BALB/c nude mice (aged 6–8 weeks, weighing 20 ± 2 g) (SPF (Beijing) biotechnology Co., Ltd., Beijing, China) were used for the in vivo assessment of the C4s’ antitumor activity. All animal experimental procedures were carried out in accordance with the standards established in the Guide for the Care and Use of Laboratory Animals published by the Institute of Laboratory Animal Resources of the National Research Council (United States) and were also approved by the Animal Laboratory of Laboratory Animal Center, Academy of Military Medical Sciences (IACUC-DWZX-2025-648) on 25 September 2025. All the animals were raised at proper temperatures and had free access to water and standard chow. MCF-7 cells (1 × 10^5^) suspended in 100 μL PBS were injected subcutaneously into the animals’ right flanks. The treatments were begun after the average tumor volume had reached 80–100 mm^3^. Intra-tumor injection (0.8 mg/kg) was administered daily for 14 days. The mice were sacrificed after the last treatment administration. Tumor tissues were isolated, photographed, and weighed. The main organ and tumor tissues were removed and further analyzed. The tumor volumes [V = (a × b^2^)/2 (V is the tumor volume, a is the tumor’s long diameter, and b is the tumor’s short diameter)] were measured with a caliper. The heart, liver, spleen, lung, kidney, and tumor were fixed in 4% paraformaldehyde for hematoxylin and eosin (H&E) staining.

### 4.16. Statistical Analysis

All data were obtained from at least 3 independent experiments and are presented as mean ± standard deviation. SPSS 26.0 software was used for statistical testing, and GraphPad Prism 9.0.0 software was used for plotting. The *t*-test was used to compare the two groups. Comparisons among multiple groups were analyzed using one-way ANOVA followed by Dunnett’s test. *p* < 0.05 was considered statistically significant.

## 5. Conclusions

In conclusion, our study has successfully developed a novel aptamer–PROTAC molecular scaffold and identified C4 as a highly potent candidate. By conjugating the aptamer AS1411 with a CRBN-targeting E3 ligand, lenalidomide, C4 effectively triggers NCL degradation and exerts remarkable antitumor effects. This study provides a new research paradigm and candidate molecule for the integration of nucleic acid aptamers with PROTAC technology, thereby expanding the molecular toolbox for PROTAC design.

## Figures and Tables

**Table 1:** AS1411-PROTAC sequences and molecular weights.

NO.	Sequence (5′-3′)	MW (Target)	MW (Observed)
AS1411	GGTGGTGGTGGTTGTGGTGGTGGTGG	8271.9	8268.1
C1	C_22_H_28_N_6_O_5_*GGTGGTGGTGGTTGTGGTGGTGGTGG	8861.2	8860.6
C2	C_24_H_32_N_6_O_5_*GGTGGTGGTGGTTGTGGTGGTGGTGG	8889.2	8888.5
C3	C_22_H_29_N_7_O_4_*GGTGGTGGTGGTTGTGGTGGTGGTGG	8860.2	8859.9
C4	C_24_H_33_N_7_O_4_*GGTGGTGGTGGTTGTGGTGGTGGTGG	8887.6	8887.8

* Represents 5′ terminal alkyne modification.

## Data Availability

The original contributions presented in this study are included in the article/Supplementary Materials. Further inquiries can be directed to the corresponding authors.

## Supplementary Materials

The following supporting information can be downloaded at: https://www.mdpi.com/article/10.3390/ph18121867/s1; Synthesis and characterization of compounds; Scheme S1: A halogen-thiol-based synthetic strategy; Scheme S2: A synthetic strategy based on halogenated acetyl bromide-thiol; Scheme S3: A synthetic strategy based on bromobenzene-thiol. Scheme S4: A synthetic strategy based on N-hydroxy-succinimidyl esters (NHS-Esters) and amines; Table S1: Schematic diagram of the synthesis strategy of AS1411-lenalidomide targeting degraded chimeras; Figure S1: MOLDI-TOF-MS spectrum of C1, C2, C3, and C4; Figure S2: The characterizations of AS1411-PROTACs; Figure S3: CCK-8 assay to detect the inhibitory effect of C4 on MCF-7 cells; Figure S4: The sigmoidal curves responding to the Tm values of AS1411 and C4; Figure S5: Cell scratch assay was used to detect the effect of AS1411 and C4 on the migration of MCF-7 cells; Figure S6: The cell cycle of MCF-7 cells treated with AS1411 and C4 detected by flow cytometry; Figure S7: Representative fluorescence histograms of the AS1411 and C4 uptake pathway in HeLa, BEAS-2B and Vero cells; Figure S8: A confocal laser scanning microscope was used to observe the fluorescence of AS1411 and C4 uptake by BEAS-2B cells.
