# Supplementary material for: Synthesis and Evaluation of AS1411-Lenalidomide-Targeted Degradation Chimera in Antitumor Therapy
Source: Pharmaceuticals (Basel). 2025 Dec 7;18(12):1867. doi: 10.3390/ph18121867 (PMC12736311; doi:10.3390/ph18121867)
Supplement: Supplementary file 1 [file pharmaceuticals-18-01867-s001.zip › pharmaceuticals-3970281-supplementary.pdf]

pharmaceuticals

Supplementary Materials: Synthesis and Evaluation of AS1411-Lenalidomide Targeted Degradation Chimera in Antitumor Therapy

Xueling Ma, Shuangshuang Liu, Xiao Dong, Xiuhua Li, Feiyan Wang, Jiawei Zhang, Zhenfang Xu, Weiguo Shi, Aiping Zheng, Aiping Zhang, Xuesong Feng, Liang Xu

Synthesis of compounds

Compound 1a. 2 g 3-(4-hydroxy-1-oxo-1,3-dihydroisoindolin-2-yl)piperidine-2,6-dione (0.0077 mol, 1 eq) was dissolved in 40 mL DMF. Then 2.22 g *tert*-butyl 6-bromohexanoate (0.0092 mol, 1.2 eq) and 3.2 g K_2CO_3 (0.0231 mol, 3 eq) were added, and the mixture was stirred at 80 °C for 24 h. After reaction, the solution was diluted with 20 mL H_2O, extracted three times with 50 mL EtOAc, and the combined organic layer was washed three times with 40 mL saturated NaCl, then dried over anhydrous Na_2SO_4 overnight. After filtration, vacuum concentration and drying, the crude product was purified by fast column chromatography (DCM : MeOH = 50 : 1). Concentration and drying gave white powder product 1a (yield 75%).

^{1}H NMR (600 MHz, DMSO - d_6): δ 10.97 (s, 1H), 7.47 (t, J = 7.8 Hz, 1H), 7.19 (dd, J = 7.5, 0.8 Hz, 1H), 7.02 (dd, J = 8.0, 0.9 Hz, 1H), 5.11 (dd, J = 13.4, 5.2 Hz, 1H), 4.36 (d, J = 17.2 Hz, 1H), 4.21 (d, J = 17.3 Hz, 1H), 4.11 (t, J = 6.3 Hz, 2H), 3.04 – 2.87 (m, 2H), 2.77 – 2.72 (m, 1H), 2.62 – 2.55 (m, 1H), 2.41 – 2.36 (m, 1H), 2.00 (dd, J = 13.1, 4.5 Hz, 1H), 1.74 (p, J = 6.5 Hz, 2H), 1.56 (p, J = 7.3 Hz, 2H), 1.38 (d, J = 2.5 Hz, 9H), 1.31 – 1.19 (m, 2H).

ESI-MS, $[C_{23}H_{30}N_2O_6]^+$. Theoretical value: 430.21. Measured value: 453.19 (M + Na^+).

^{1}H NMR of compound **1a**

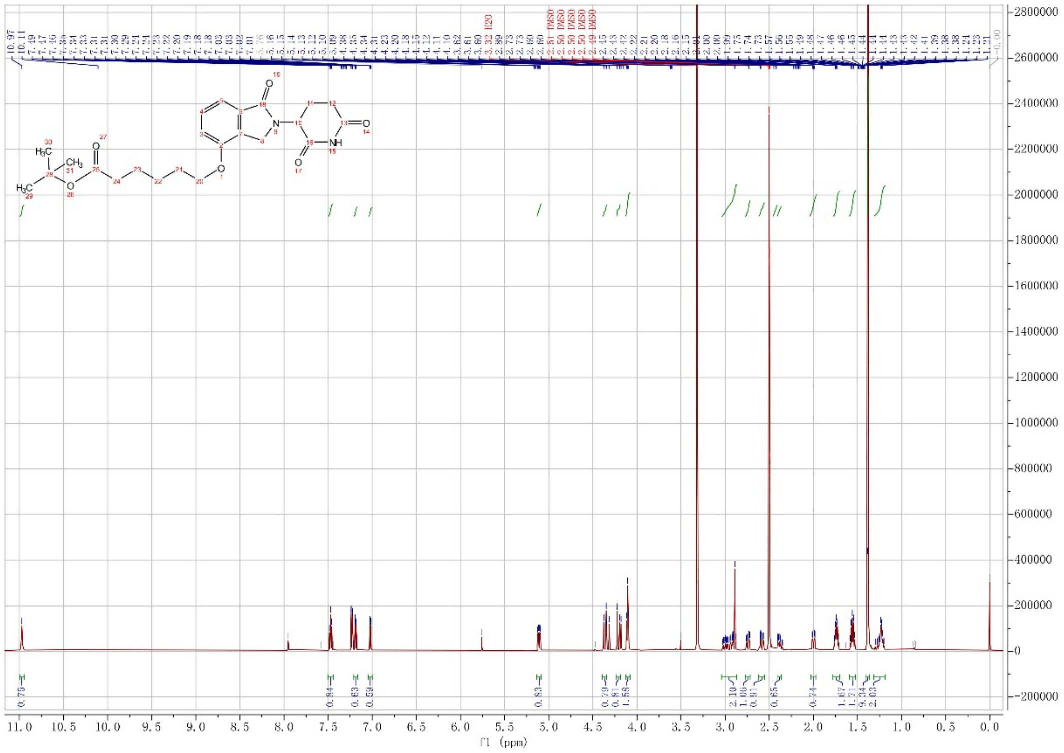

ESI-MS of compound **1a**

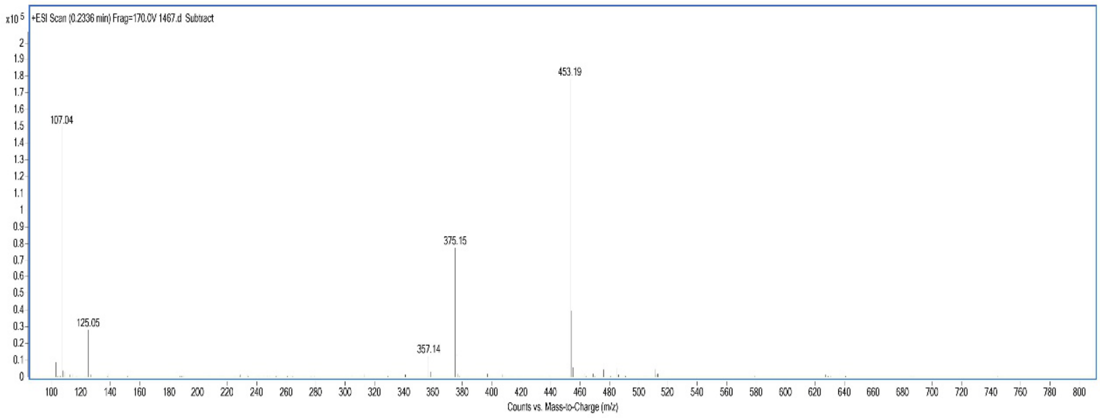

Compound 1b. 0.5 g 1a (0.0012 mol, 1 eq) was dissolved in 2 mL DCM. 6 g TFA (0.0524 mol, 45 eq) was added, and the mixture was stirred at room temperature for 2 h. After reaction, the solvent was evaporated, 5 mL Et_2O was added. The mixture was filtered, and the precipitate was collected to obtain white powder product 1b (yield 99 %).

^{1}H NMR (600 MHz, DMSO - d_6): δ 11.00 (s, 1H), 7.28 (t, J = 7.7 Hz, 1H), 6.92 (d, J = 7.4 Hz, 1H), 6.74 (d, J = 8.0 Hz, 1H), 5.11 (dd, J = 13.3, 5.1 Hz, 1H), 4.22 (d, J = 17.0 Hz, 1H), 4.12 (d, J = 17.1 Hz, 1H), 4.03 (q, J = 6.4, 5.8 Hz, 2H), 2.92 (ddd, J = 17.3, 13.6, 5.4 Hz, 1H), 2.62 (d, J = 17.5 Hz, 1H), 2.29 (dd, J = 13.3, 4.5 Hz, 1H), 2.19 (t, J = 7.3 Hz, 2H), 2.03 (ddq, J = 10.8, 5.6, 3.0, 2.5 Hz, 1H), 1.57 (p, J = 7.1 Hz, 2H), 1.52 (q, J = 7.5 Hz, 2H), 1.23 (d, J = 6.0 Hz, 2H).

ESI-MS, $[C_{19}H_{22}N_2O_6]^+$. Theoretical value: 374.15. Measured value: 375.15 (M + H^+).

^{1}H NMR of compound **1b**

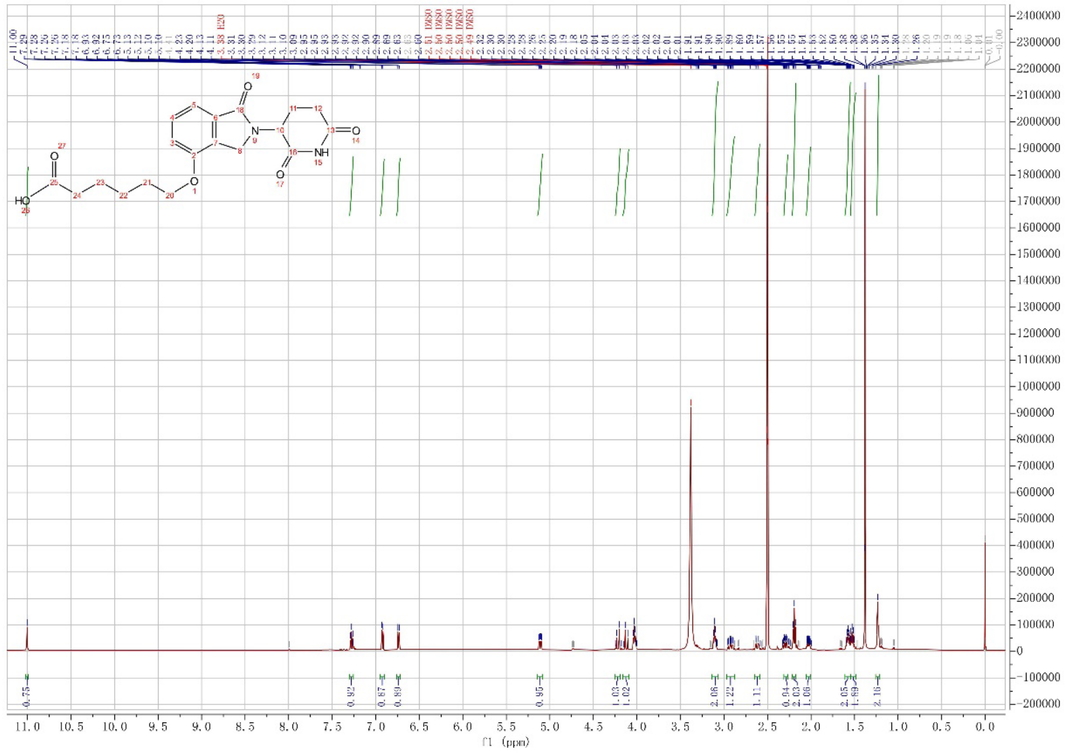

ESI-MS of compound **1b**

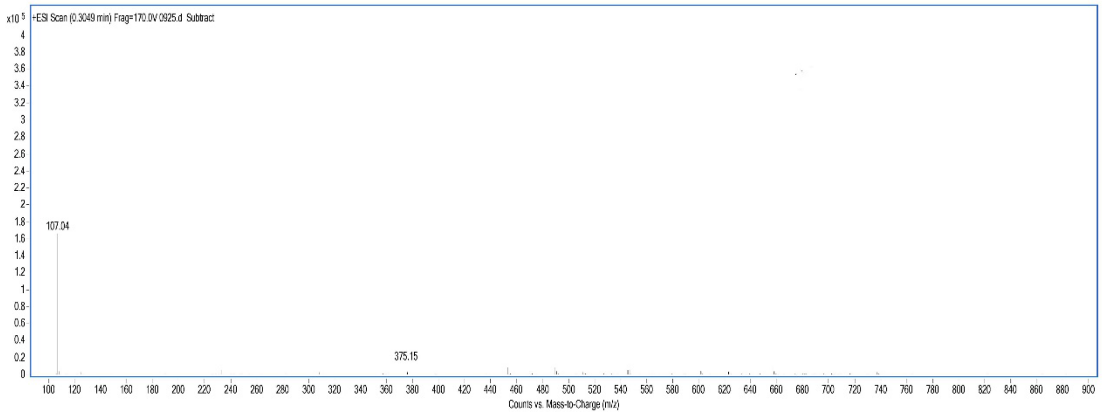

Compound 1c. Solution A: 0.1 g 1b (0.00027 mol, 1 eq) was dissolved in 3 mL DMF. Then 0.102 g HBTU (0.00027 mol, 1 eq), 0.036 g HOBt (0.00027 mol, 1 eq), and 0.172 g DIPEA (0.0013 mol, 5 eq) were added. Solution B: 0.027 g 3-azidopropylamine (0.00027 mol, 1 eq) was mixed with TFA ($V_{3\text{-axidopropylamine}}$: V_TFA = 1 : 1). Solution A was stirred for 10 min before adding Solution B. The reaction mixture was stirred at room temperature for 12 h. The crude product was purified by thin-layer chromatography (DCM : MeOH = 10 : 1) to obtain light yellow powder product 1c (yield 43 %).

^{1}H NMR (600 MHz, DMSO - d_6): δ 11.01 (s, 1H), 7.87 (t, J = 5.6 Hz, 1H), 7.28 (t, J = 7.7 Hz, 1H), 6.92 (d, J = 7.4 Hz, 1H), 6.73 (d, J = 8.1 Hz, 1H), 5.11 (dd, J = 13.3, 5.1 Hz, 1H), 4.23 (d, J = 17.1 Hz, 1H), 4.12 (d, J = 17.1 Hz, 1H), 3.75 (t, J = 5.5 Hz, 1H), 3.58 (d, J = 18.2 Hz, 3H), 3.09 (dq, J = 12.8, 6.6 Hz, 5H), 2.94 (q, J = 4.9, 4.5 Hz, 1H), 2.93 – 2.89 (m, 1H), 2.65 – 2.59 (m, 1H), 2.29 (qd, J = 13.0, 4.6 Hz, 1H), 2.07 (t, J = 7.4 Hz, 1H), 2.05 – 1.97 (m, 1H), 1.63 (p, J = 6.8 Hz, 2H), 1.55 (dp, J = 26.0, 7.4 Hz, 3H).

ESI-MS, $[C_{22}H_{28}N_6O_5]^+$. Theoretical value: 456.21. Measured value: 479.20 (M + Na^+).

HRMS, $[C_{22}H_{28}N_6O_5]^+$. Theoretical value: 456.2121. Measured value: 457.2191 & 479.2012 (M + H^+ & M + Na^+).

^{1}H NMR of compound **1c**

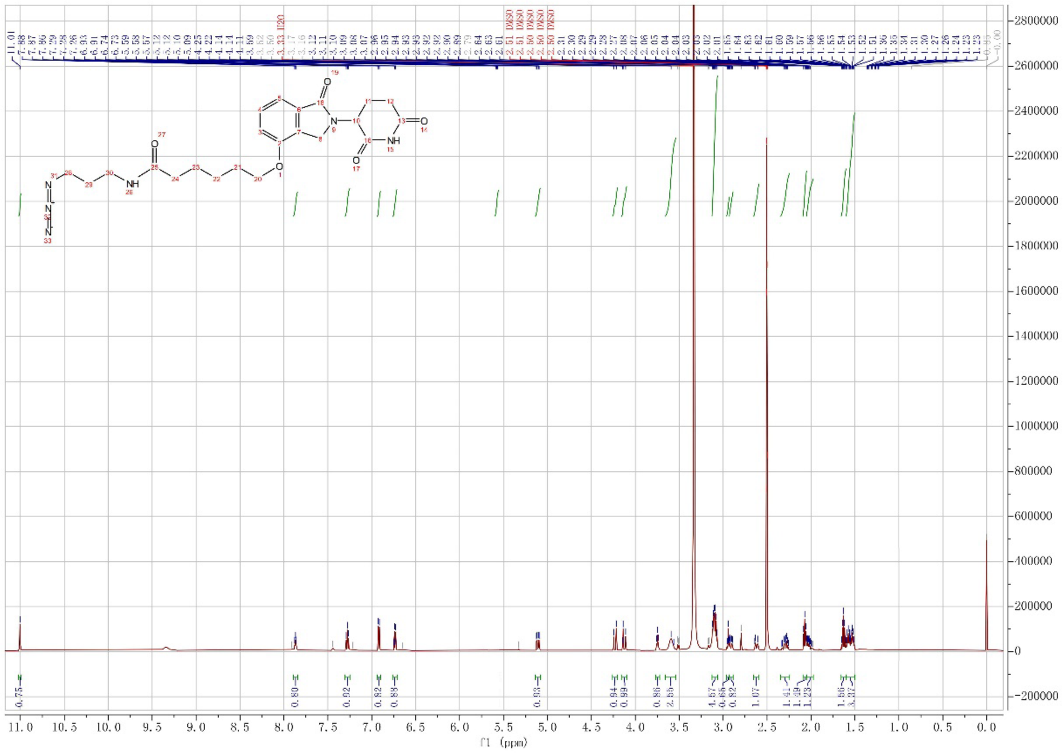

ESI-MS of compound **1c**

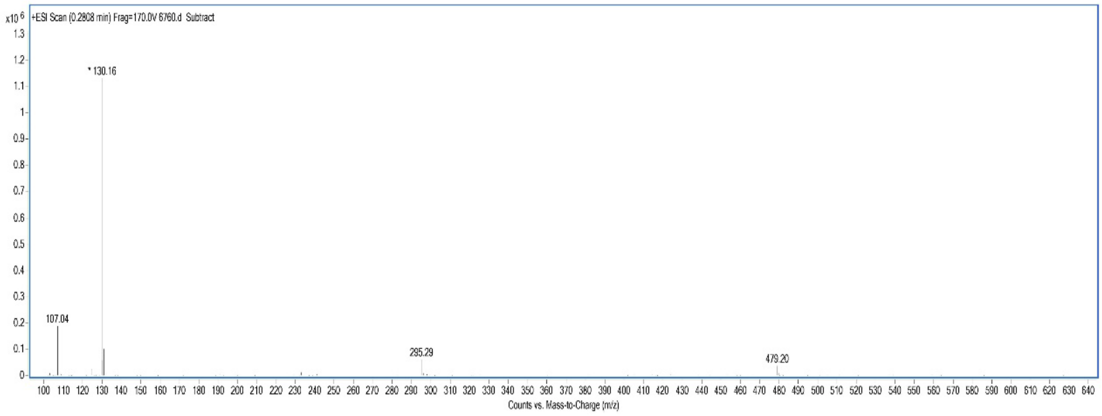

HRMS of compound **1c**

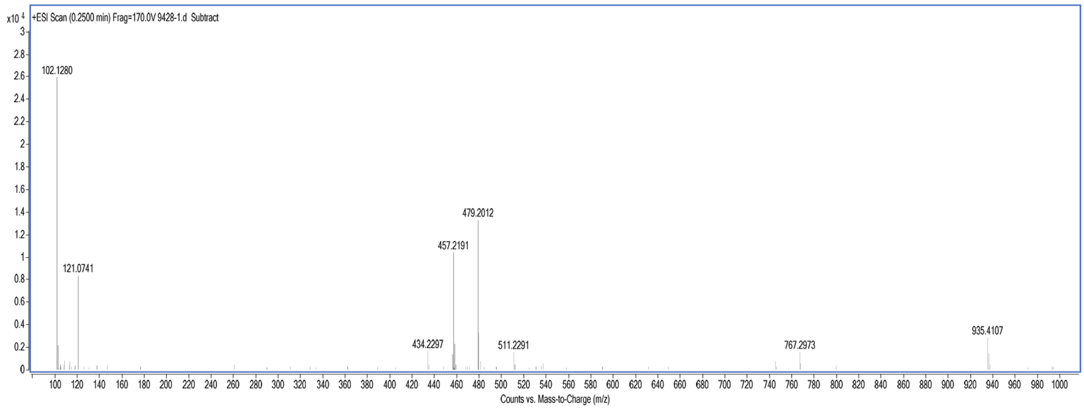

Compound 2a. 2 g 3-(4-hydroxy-1-oxo-1,3-dihydroisoindolin-2-yl)piperidine-2,6-dione (0.0077 mol, 1 eq) was dissolved in 40 mL DMF. Then 2.58 g *tert*-butyl 8-bromooctanoate (0.0092 mol, 1.2 eq) and 3.2 g K_2CO_3 (0.0231 mol, 3 eq) were added, and the mixture was stirred at 80 °C for 24 h. Subsequent operations were consistent with the procedure for 1a. White powder product 2a was obtained (yield 45 %).

^{1}H NMR (600 MHz, DMSO - d_6): δ 10.96 (s, 1H), 7.47 (t, J = 7.8 Hz, 1H), 7.19 (dd, J = 7.4, 0.9 Hz, 1H), 7.02 (dd, J = 8.0, 0.9 Hz, 1H), 5.10 (dd, J = 13.3, 5.1 Hz, 1H), 4.35 (dd, J = 20.1, 17.2 Hz, 1H), 4.24 – 4.16 (m, 1H), 4.11 (t, J = 6.4 Hz, 2H), 3.00 (ddd, J = 17.3, 13.7, 5.4 Hz, 1H), 2.95 – 2.86 (m, 1H), 2.77 – 2.71 (m, 1H), 2.58 (d, J = 17.3 Hz, 1H), 2.45 (dd, J = 13.1, 4.5 Hz, 1H), 2.16 (dt, J = 11.4, 7.3 Hz, 2H), 2.03 – 1.97 (m, 1H), 1.73 (p, J = 6.5 Hz, 2H), 1.38 (d, J = 1.5 Hz, 10H), 1.30 – 1.22 (m, 5H). ESI-MS, $[C_{25}H_{34}N_2O_6]^+$. Theoretical value: 458.24. Measured value: 481.22 (M + Na^+).

^{1}H NMR of compound **2a**

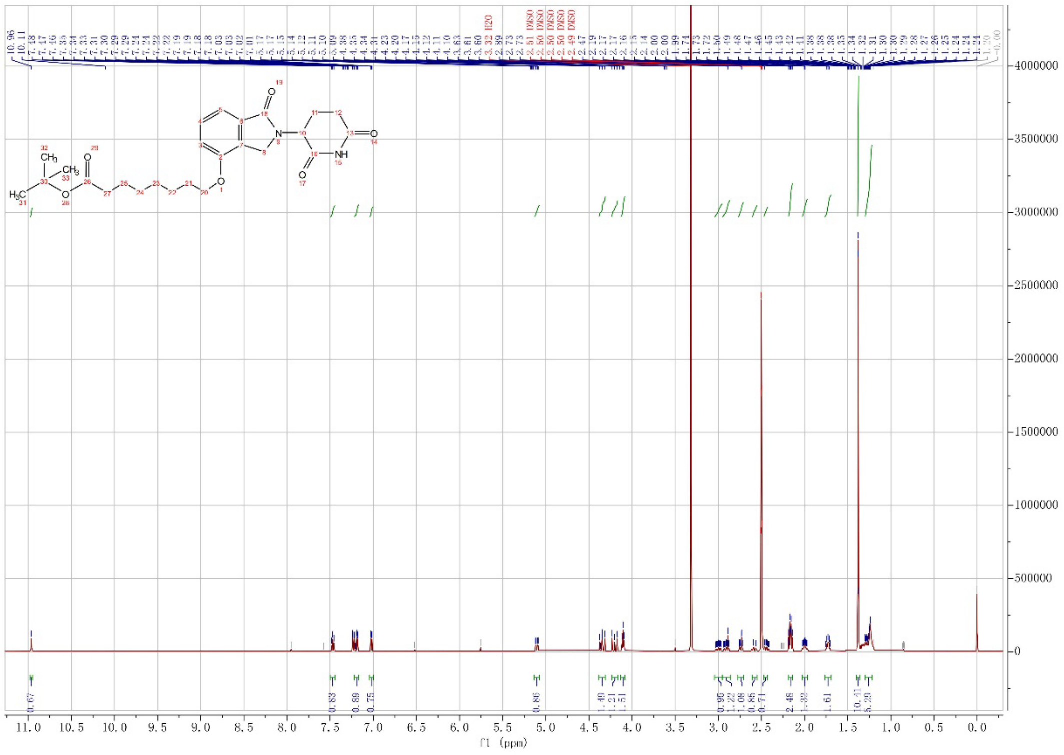

ESI-MS of compound **2a**

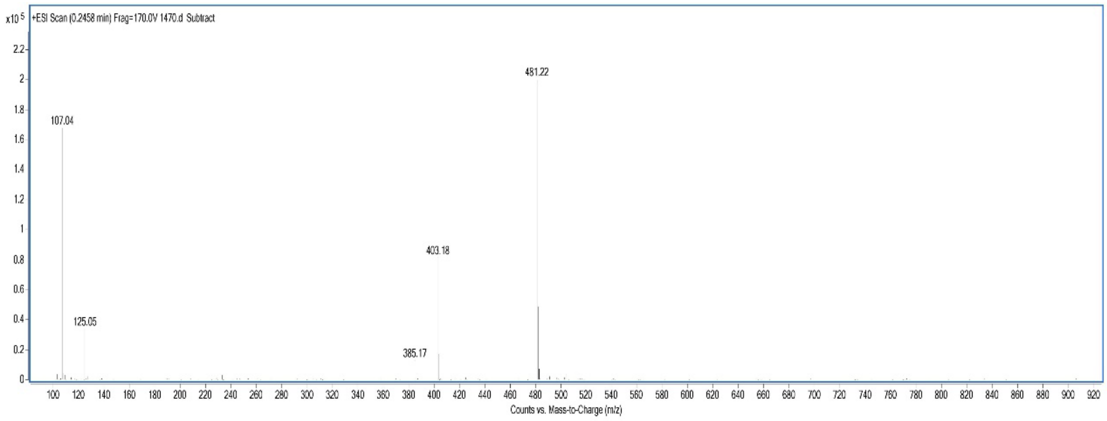

Compound 2b. 0.5 g 2a (0.0011 mol, 1 eq) was dissolved in 2 mL DCM. 5.61 g TFA (0.0492 mol, 45 eq) was added, and the mixture was stirred at room temperature for 2 h. Subsequent operations were consistent with the procedure for 1b. White powder product 2b was obtained (yield 98 %).

^{1}H NMR (600 MHz, DMSO - d_6): δ 11.00 (s, 1H), 7.28 (t, J = 7.7 Hz, 1H), 6.95 – 6.89 (m, 1H), 6.74 (d, J = 8.0 Hz, 1H), 5.11 (dd, J = 13.3, 5.2 Hz, 1H), 4.22 (d, J = 17.1 Hz, 1H), 4.12 (d, J = 17.1 Hz, 1H), 4.01 (q, J = 6.6 Hz, 2H), 2.92 (ddd, J = 17.3, 13.6, 5.4 Hz, 1H), 2.62 (ddd, J = 17.3, 4.5, 2.4 Hz, 1H), 2.29 (qd, J = 13.3, 4.4 Hz, 1H), 2.03 (dtd, J = 12.6, 5.2, 2.2 Hz, 1H), 1.94 – 1.86 (m, 2H), 1.57 (p, J = 7.2 Hz, 2H), 1.49 (p, J = 7.3 Hz, 2H), 1.37 – 1.32 (m, 2H), 1.33 – 1.23 (m, 4H).

ESI-MS, $[C_{21}H_{26}N_2O_6]^+$. Theoretical value: 402.18. Measured value: 403.19 & 425.17 (M + H^+ & M + Na^+).

^{1}H NMR of compound **2b**

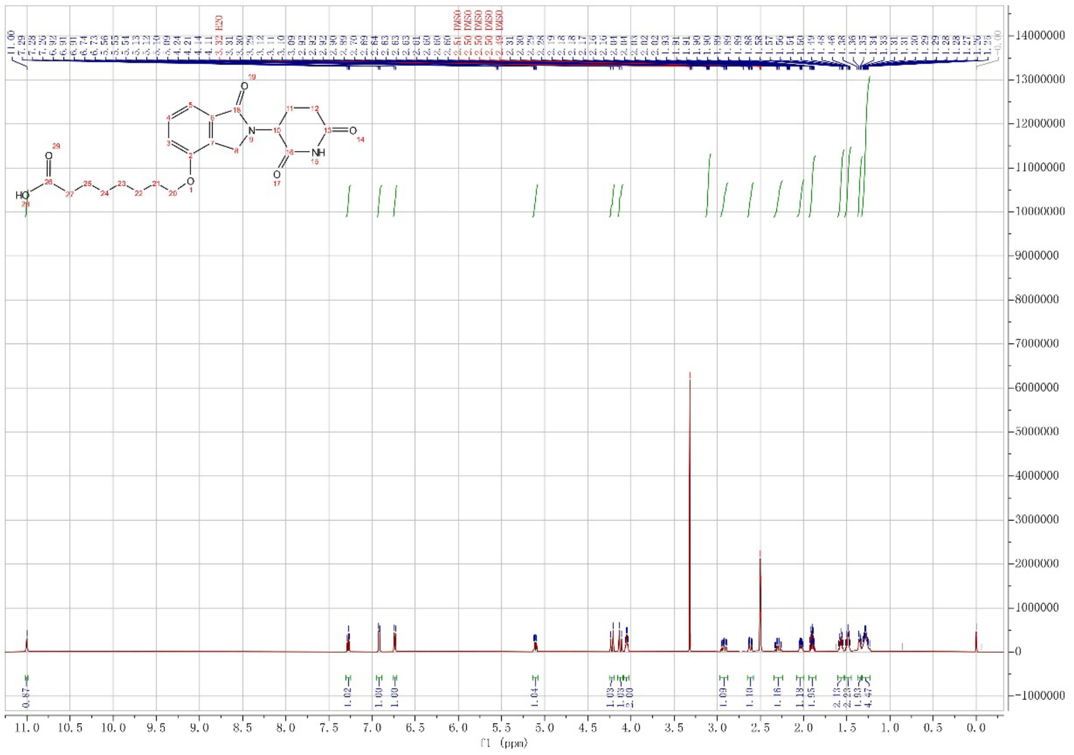

ESI-MS of compound **2b**

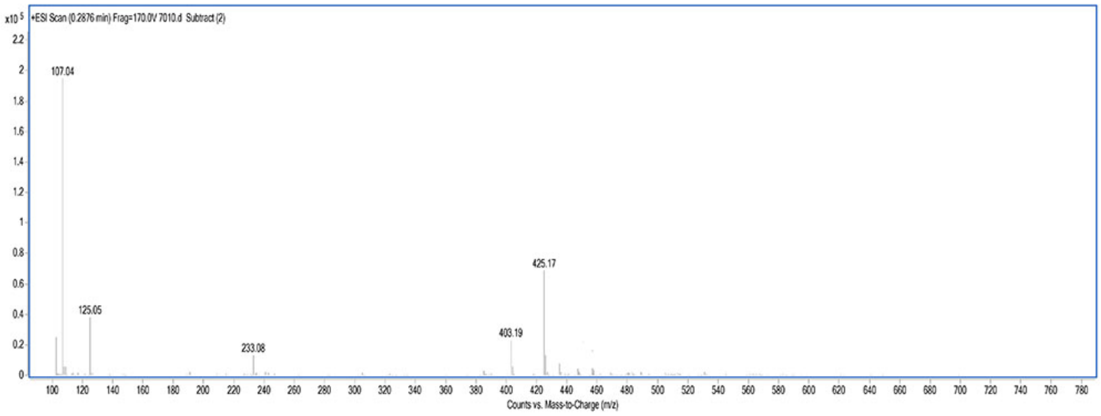

Compound 2c. Solution A: 0.1 g 2b (0.00025 mol, 1 eq) was dissolved in 3 mL DMF. Then 0.095 g HBTU (0.00025 mol, 1 eq), 0.034 g HOBt (0.00027 mol, 1 eq), and 0.162 g DIPEA (0.00125 mol, 5 eq) were added. Solution B: 0.025 g 3-azidopropylamine (0.00025 mol, 1 eq) was mixed with TFA ($V_{3-azidopropylamine}$: V_TFA = 1 : 1). Subsequent operations were consistent with the procedure for 1c. White powder product 2c was obtained (yield 40 %).

^{1}H NMR (600 MHz, DMSO - d_6): δ 11.01 (s, 1H), 7.85 (t, J = 5.8 Hz, 1H), 7.29 (t, J = 7.7 Hz, 1H), 6.93 (d, J = 7.4 Hz, 1H), 6.74 (d, J = 8.1 Hz, 1H), 5.10 (dd, J = 13.3, 5.1 Hz, 1H), 4.26 – 4.17 (m, 1H), 4.12 (d, J = 17.1 Hz, 1H), 4.05 (t, J = 6.8 Hz, 2H), 3.14 – 3.05 (m, 4H), 2.92 (ddd, J = 17.4, 13.6, 5.5 Hz, 1H), 2.66 – 2.59 (m, 1H), 2.32 (qd, J = 13.2, 4.4 Hz, 1H), 2.04 – 2.01 (qd, J = 13.2, 4.4 Hz, 1H), 1.63 (p, J = 6.8 Hz, 2H), 1.57 (p, J = 7.2 Hz, 2H), 1.49 (p, J = 7.4 Hz, 2H), 1.38 – 1.22 (m, 8H).

^{13}C NMR (150 MHz, DMSO - d_6): δ 172.81, 172.14, 168.70, 162.85, 154.30, 133.64, 130.14, 124.37, 115.54, 114.96, 68.39, 52.69, 49.03, 45.56, 38.64, 36.20, 35.81, 31.89, 31.66, 31.19, 28.99, 28.87, 26.70, 25.65.

ESI-MS, $[C_{24}H_{32}N_6O_5]^+$. Theoretical value: 484.24. Measured value: 507.23 (M + Na^+).

HRMS, $[C_{24}H_{32}N_6O_5]^+$. Theoretical value: 484.2434. Measured value: 507.2328 (M + Na^+).

^{1}H NMR of compound **2c**

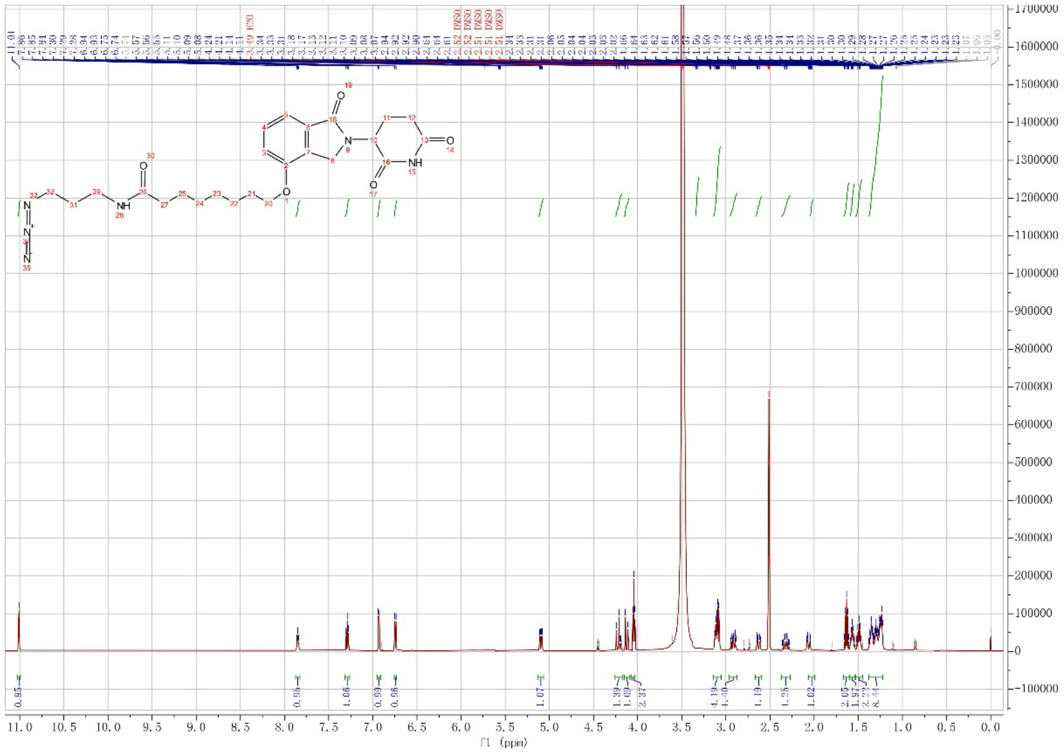

^{13}C NMR of compound **2c**

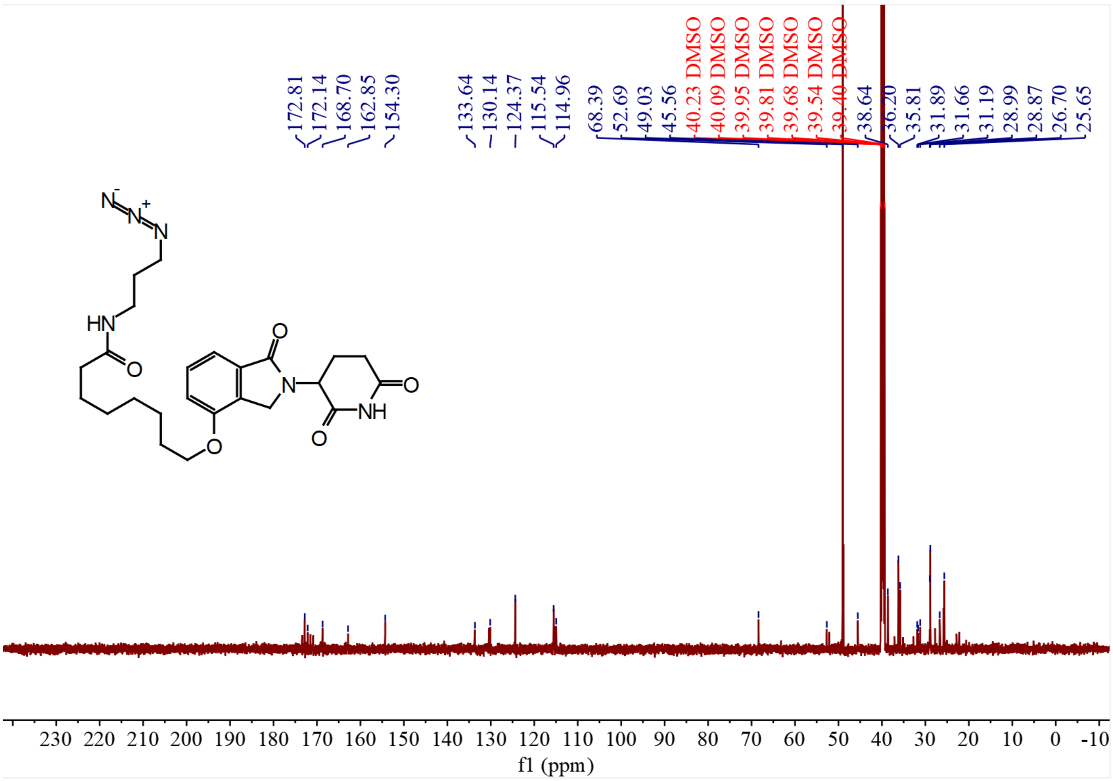

ESI-MS of compound **2c**

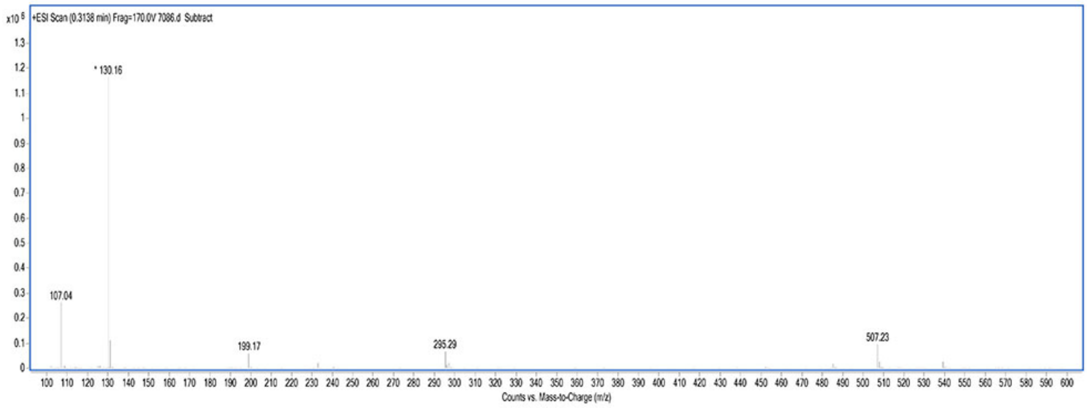

HRMS of compound **2c**

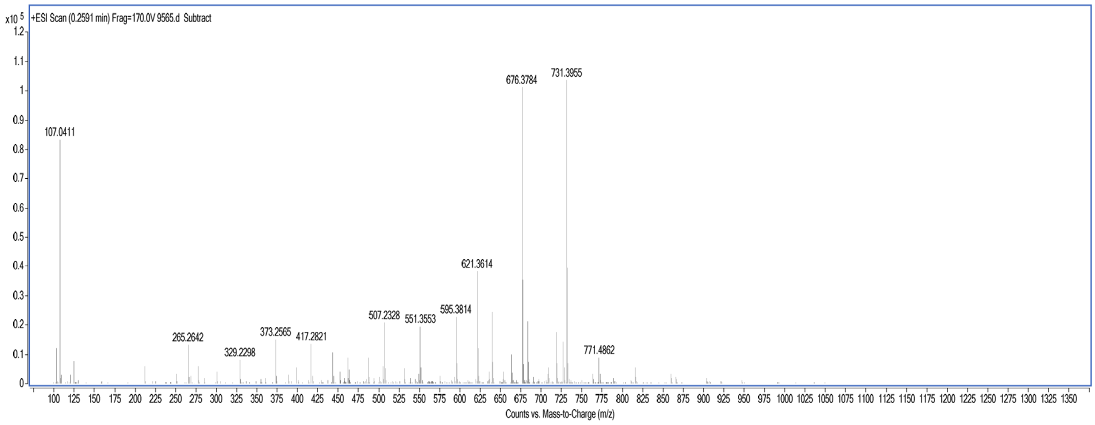

Compound 3a. 2 g lenalidomide (0.0077 mol, 1 eq) was dissolved in 30 mL NMP. Then 2.32 g *tert*-butyl 6-bromohexanoate (0.0090 mol, 1.2 eq) and 3 g DIPEA (0.0232 mol, 3 eq) were added. The mixture was stirred at 120 °C for 15 h. After reaction, the solution was diluted with 20 mL H_2O, extracted three times with 40 mL EtOAc. The combined organic layer was washed three times with 40 mL saturated NaCl, dried over anhydrous Na_2SO_4 overnight. After filtration and vacuum concentration, the crude product was purified by fast column chromatography (DCM : MeOH = 50 : 1) to obtain pale yellow powder product 3a (yield 40 %).

^{1}H NMR (600 MHz, DMSO - d_6): δ 11.00 (s, 1H), 7.28 (d, J = 7.7 Hz, 1H), 6.92 (d, J = 7.4 Hz, 1H), 6.74 (d, J = 8.0 Hz, 1H), 5.56 (s, 1H), 5.11 (dd, J = 13.3, 5.1 Hz, 1H), 4.22 (d, J = 17.0 Hz, 1H), 4.12 (d, J = 17.1 Hz, 1H), 3.10 (q, J = 6.4, 5.8 Hz, 2H), 2.92 (ddd, J = 17.3, 13.6, 5.4 Hz, 1H), 2.69 (s, 1H), 2.62 (d, J = 17.5 Hz, 1H), 2.29 (dd, J = 13.3, 4.5 Hz, 1H), 2.19 (s, 1H), 2.03 (ddq, J = 10.8, 5.6, 3.0, 2.5 Hz, 1H), 1.58 (q, J = 7.3 Hz, 2H), 1.52 (q, J = 7.5 Hz, 2H), 1.38 (s, 10H), 1.34 (s, 1H).

ESI-MS, $[C_{23}H_{31}N_3O_5]^+$. Theoretical value: 429.23. Measured value: 452.20 (M + Na^+).

^{1}H NMR of compound **3a**

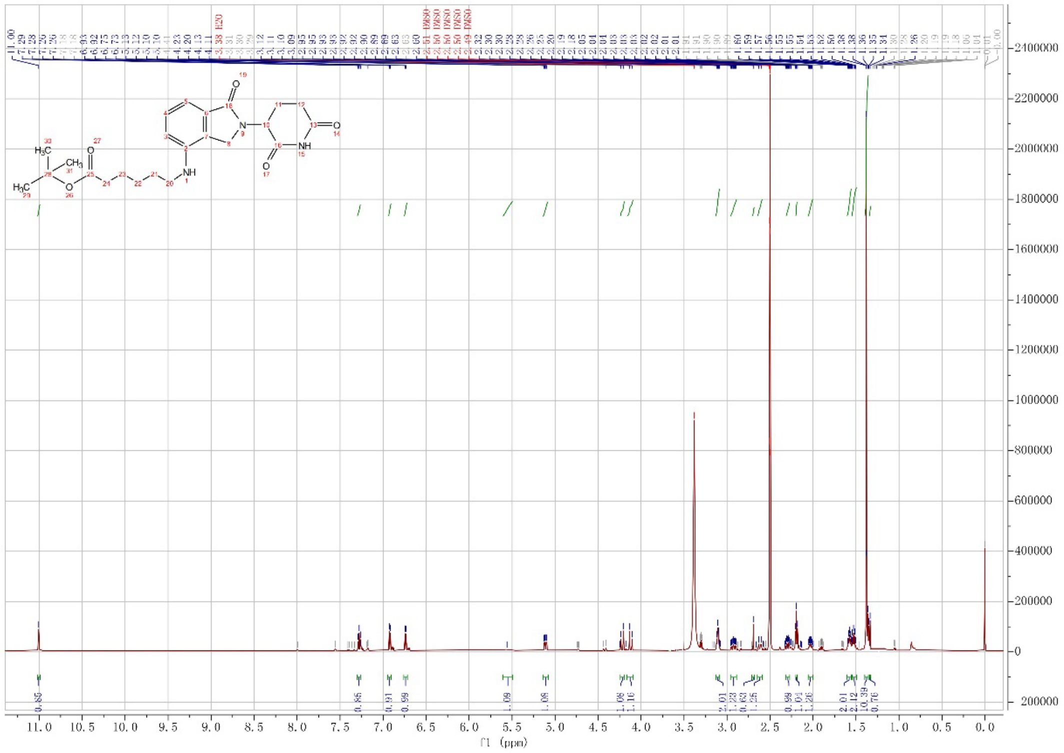

ESI-MS of compound **3a**

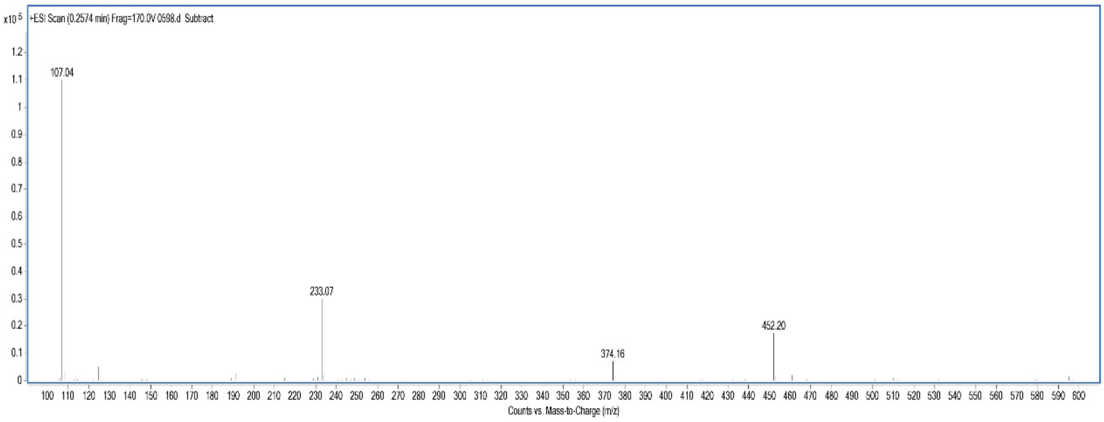

Compound 3b. 0.5 g 3a (0.0012 mol, 1 eq) was dissolved in 2 mL DCM. 6 g TFA (0.0524 mol, 45 eq) was added, and the mixture was stirred at room temperature for 2 h. After reaction, the solvent was evaporated, 5 mL Et_2O was added. The mixture was filtered, and the precipitate was collected to obtain pale yellow powder product 3b (yield 99 %).

^{1}H NMR (600 MHz, DMSO - d_6) δ 11.01 (s, 1H), 7.28 (t, J = 7.7 Hz, 1H), 6.93 (d, J = 7.4 Hz, 1H), 6.75 (d, J = 8.0 Hz, 1H), 5.11 (dd, J = 13.3, 5.1 Hz, 1H), 4.23 (d, J = 17.1 Hz, 2H), 4.13 (d, J = 17.1 Hz, 2H), 3.38 (q, J = 7.0 Hz, 1H), 3.11 (t, J = 7.1 Hz, 2H), 2.92 (ddd, J = 17.3, 13.6, 5.4 Hz, 1H), 2.62 (dt, J = 17.2, 3.8 Hz, 1H), 2.30 (qd, J = 13.2, 4.5 Hz, 1H), 2.22 (t, J = 7.4 Hz, 2H), 1.56 (dp, J = 23.0, 7.4 Hz, 3H), 1.40 – 1.36 (m, 1H), 1.09 (t, J = 7.0 Hz, 1H).

ESI-MS, $[C_{19}H_{23}N_3O_5]^+$. Theoretical value: 373.16. Measured value: 374.15 & 396.13 (M + H^+ & M + Na^+).

^{1}H NMR of compound **3b**

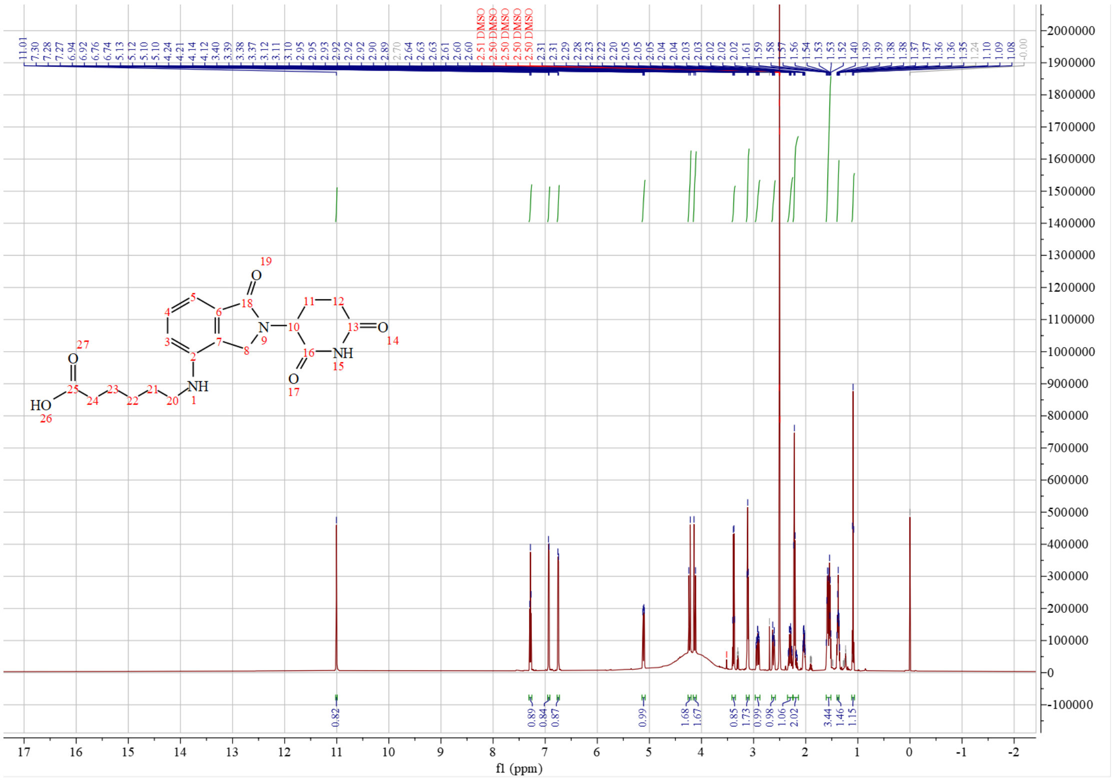

ESI-MS of compound **3b**

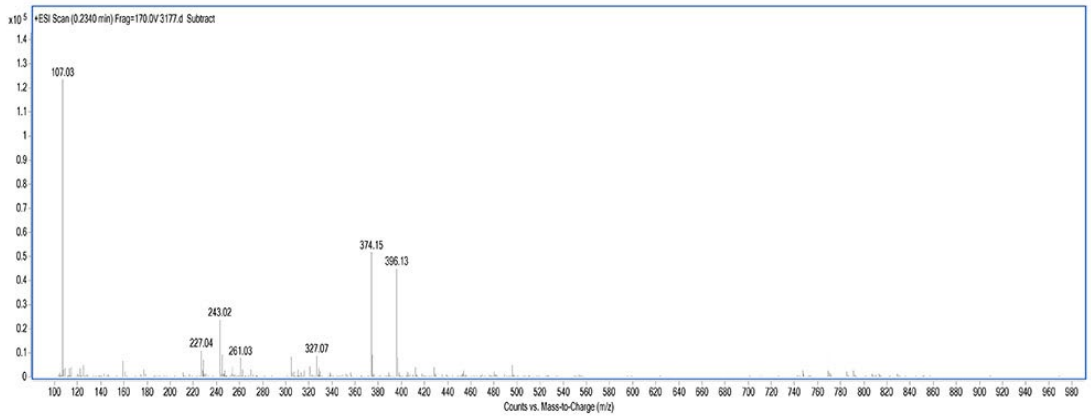

Compound 3c. Solution A: 0.1 g 3b (0.00026 mol, 1 eq) was dissolved in 3 mL DMF. Then 0.102 g HBTU (0.00026 mol, 1 eq), 0.036 g HOBt (0.00026 mol, 1 eq), and 0.172 g DIPEA (0.0013 mol, 5 eq) were added. Solution B: 0.028 g 3 - azidopropylamine (0.00026 mol, 1 eq) was mixed with TFA ($V_{3\text{-}axi}d_{oprop}y_{lamine}$: VTFA = 1 : 1). Solution A was stirred for 10 min before adding Solution B. The reaction mixture was stirred at room temperature for 12 h. The crude product was purified by thin-layer chromatography (DCM : MeOH = 10 : 1) to obtain light yellow powder product 3c (yield 52 %).

^{1}H NMR (600 MHz, DMSO - d_6): δ 11.01 (s, 1H), 7.87 (t, J = 5.6 Hz, 1H), 7.28 (t, J = 7.7 Hz, 1H), 6.92 (d, J = 7.4 Hz, 1H), 6.73 (d, J = 8.1 Hz, 1H), 5.58 (t, J = 5.5 Hz, 1H), 5.11 (dd, J = 13.3, 5.1 Hz, 1H), 4.23 (d, J = 17.1 Hz, 1H), 4.12 (d, J = 17.1 Hz, 1H), 3.58 (d, J = 18.2 Hz, 2H), 3.09 (dq, J = 12.8, 6.6 Hz, 5H), 2.97 – 2.88 (m, 2H), 2.65 – 2.59 (m, 1H), 2.29 (qd, J = 13.0, 4.6 Hz, 2H), 2.08 (d, J = 7.4 Hz, 1H), 2.06 – 1.98 (m, 2H), 1.65 – 1.51 (m, 5H).

^{13}C NMR (150 MHz, DMSO - d_6): δ 173.38, 172.57, 171.74, 169.37, 144.23, 132.51, 129.69, 126.92, 112.18, 110.38, 51.95, 48.90, 46.19, 43.07, 36.22, 35.86, 31.72, 28.93, 28.78, 26.78, 25.60, 23.31.

ESI-MS, $[C_{22}H_{29}N_7O_4]^+$. Theoretical value: 455.23. Measured value: 456.23 & 478.21 (M + H^+ & M + Na^+).

HRMS, $[C_{22}H_{29}N_7O_4]^+$. Theoretical value: 455.2281. Measured value: 478.2173 (M + Na^+).

^{1}H NMR of compound **3c**

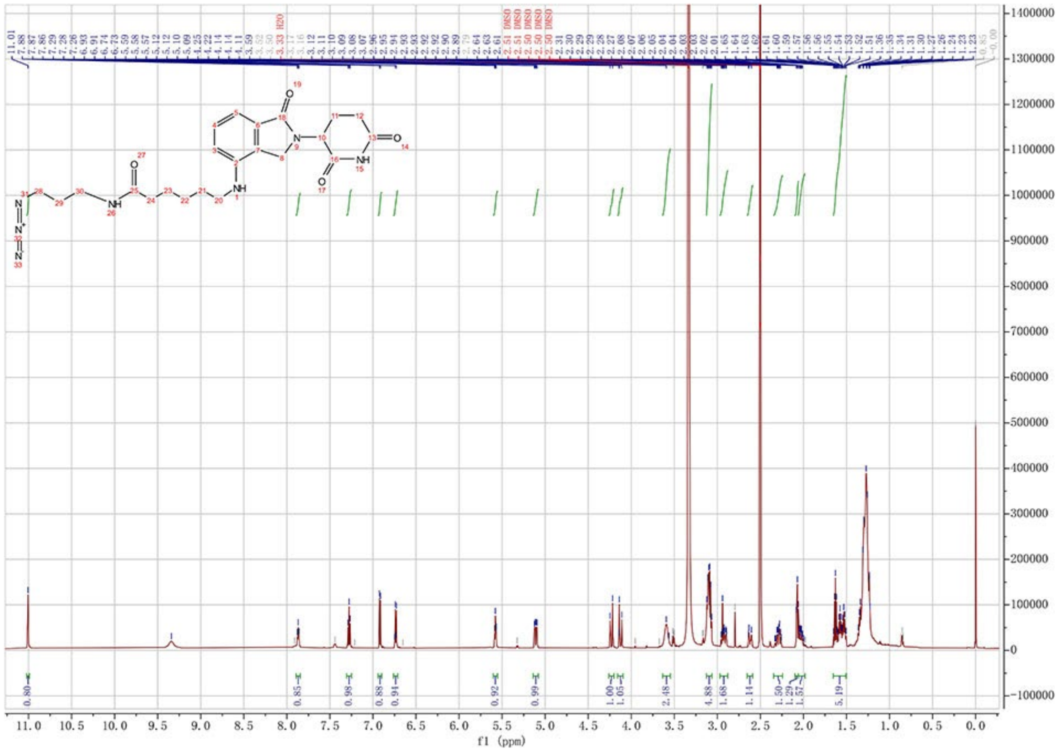

^{13}C NMR of compound **3c**

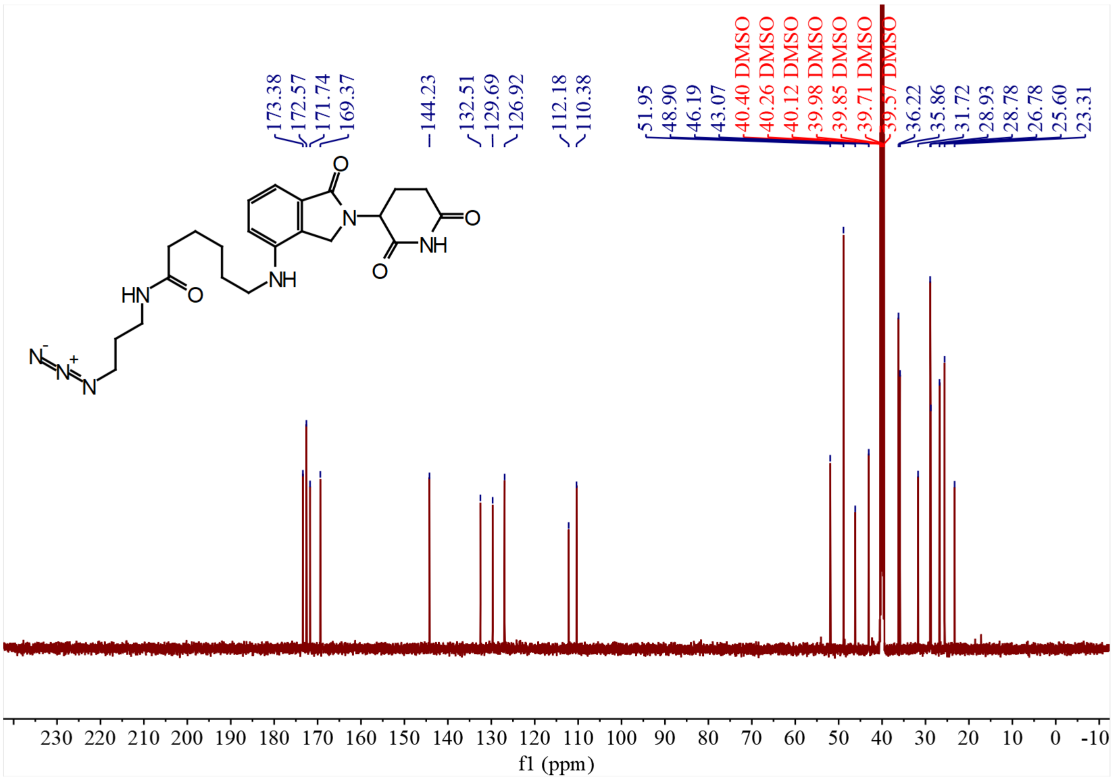

ESI-MS of compound **3c**

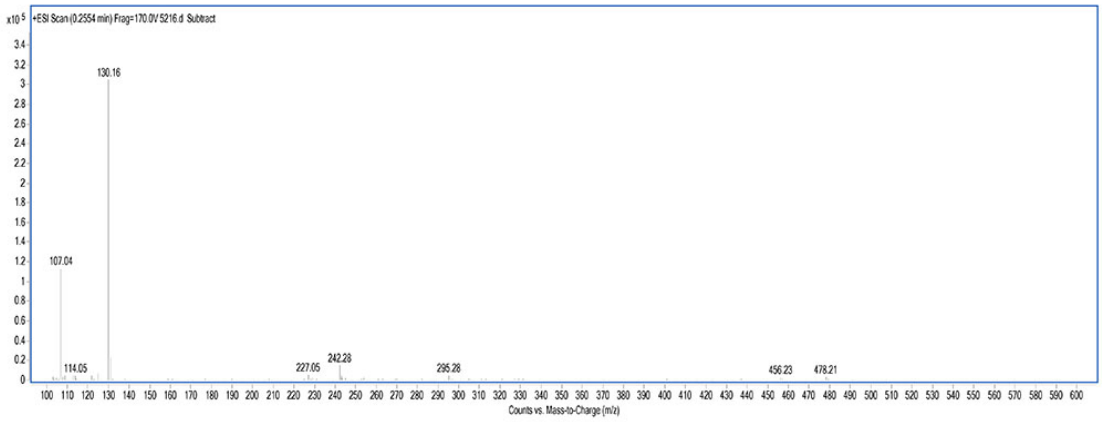

HRMS of compound **3c**

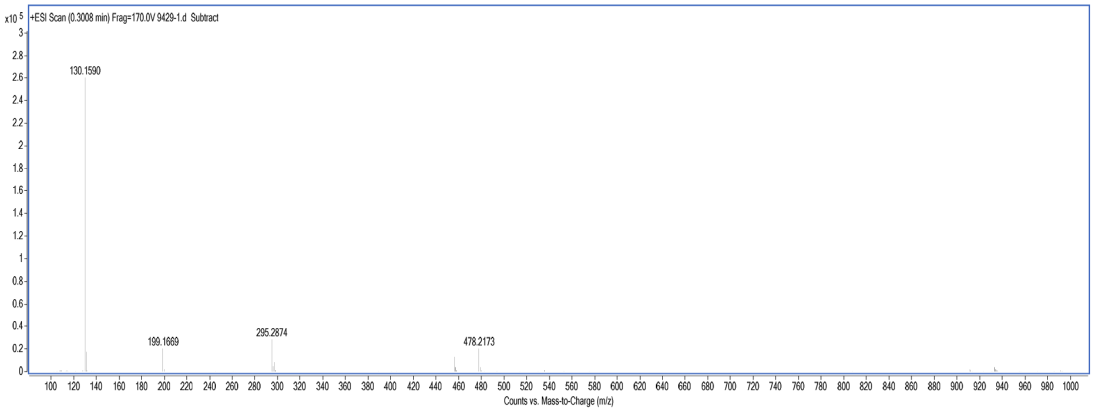

Compound 4a. 2 g lenalidomide (0.0077 mol, 1 eq) was dissolved in 30 mL NMP. Then 2.58 g *tert* - butyl 8 - bromooctanoate (0.0090 mol, 1.2 eq) and 3 g DIPEA (0.0232 mol, 3 eq) were added. The mixture was stirred at 120 °C for 15 h. Subsequent operations were consistent with the procedure for 3a. Pale yellow powder product 4a was obtained (yield 45 %). ^{1}H NMR (600 MHz, DMSO - d_6) δ 11.00 (s, 1H), 7.28 (t, J = 7.7 Hz, 1H), 6.94 – 6.90 (m, 1H), 6.74 (d, J = 8.0 Hz, 1H), 5.55 (t, J = 5.5 Hz, 1H), 5.11 (dd, J = 13.3, 5.2 Hz, 1H), 4.22 (d, J = 17.1 Hz, 1H), 4.12 (d, J = 17.1 Hz, 1H), 3.11 (q, J = 6.6 Hz, 2H), 2.92 (ddd, J = 17.3, 13.6, 5.4 Hz, 1H), 2.70 (d, J = 1.0 Hz, 2H), 2.62 (ddd, J = 17.3, 4.5, 2.4 Hz, 1H), 2.17 (d, J = 3.2 Hz, 2H), 2.03 (dtd, J = 12.6, 5.2, 2.2 Hz, 1H), 1.92 – 1.89 (m, 1H), 1.57 (p, J = 7.2 Hz, 2H), 1.49 (p, J = 7.3 Hz, 2H), 1.38 (s, 9H), 1.29 (ddt, J = 15.4, 11.7, 6.6 Hz, 4H).

ESI-MS, $[C_{25}H_{35}N_3O_5]^+$. Theoretical value: 457.26. Measured value: 480.24 (M + Na^+).

^{1}H NMR of compound **4a**

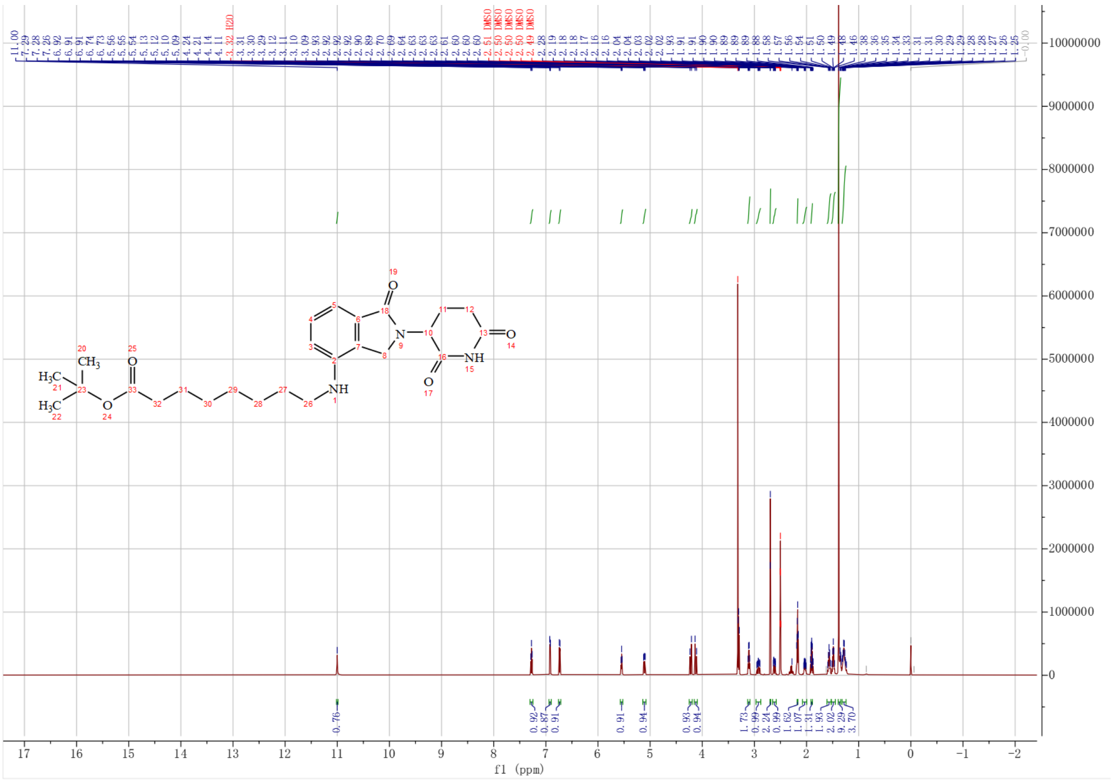

ESI-MS of compound **4a**

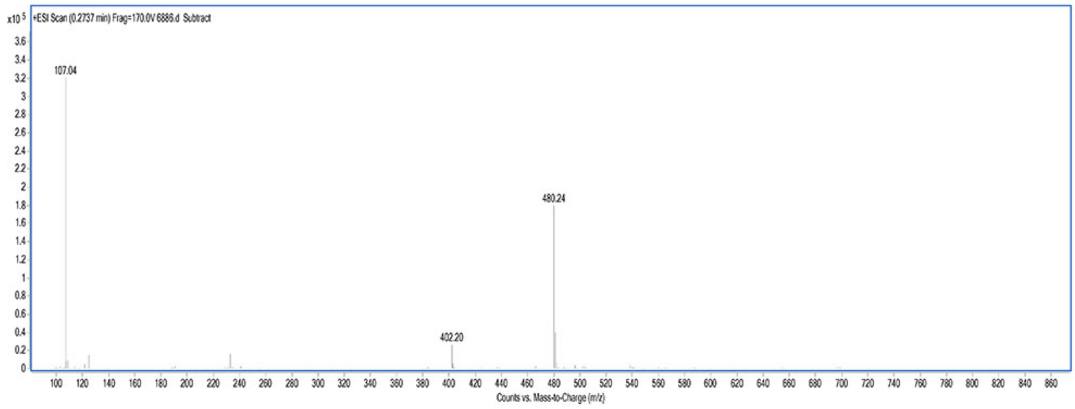

Compound 4b. 0.5 g 4a (0.0011 mol, 1 eq) was dissolved in 2 mL DCM. 5.61 g TFA (0.0492 mol, 45 eq) was added, and the mixture was stirred at room temperature for 2 h. Subsequent operations were consistent with the procedure for 3b. Pale yellow powder product 4b was obtained (yield 99 %).

^{1}H NMR (600 MHz, DMSO - d_6): δ 11.00 (s, 1H), 7.28 (t, J = 7.7 Hz, 1H), 6.92 (d, J = 7.4 Hz, 1H), 6.74 (d, J = 8.0 Hz, 1H), 5.11 (dd, J = 13.3, 5.1 Hz, 1H), 4.23 (d, J = 17.1 Hz, 1H), 4.13 (d, J = 17.1 Hz, 1H), 3.11 (t, J = 7.1 Hz, 2H), 2.92 (ddd, J = 17.3, 13.6, 5.4 Hz, 1H), 2.65 – 2.58 (m, 1H), 2.30 (qd, J = 13.2, 4.4 Hz, 1H), 2.19 (t, J = 7.4 Hz, 2H), 2.03 (dtd, J = 12.8, 5.4, 2.4 Hz, 1H), 1.57 (p, J = 7.1 Hz, 2H), 1.50 (p, J = 7.3 Hz, 2H), 1.35 (dp, J = 11.9, 6.9, 6.0 Hz, 3H), 1.29 (td, J = 7.4, 6.2, 3.3 Hz, 4H).
ESI-MS, $[C_{21}H_{27}N_3O_5]^+$. Theoretical value: 401.20. Measured value: 402.20 & 424.18 (M + H^+ & M + Na^+).

^{1}H NMR of compound **4b**

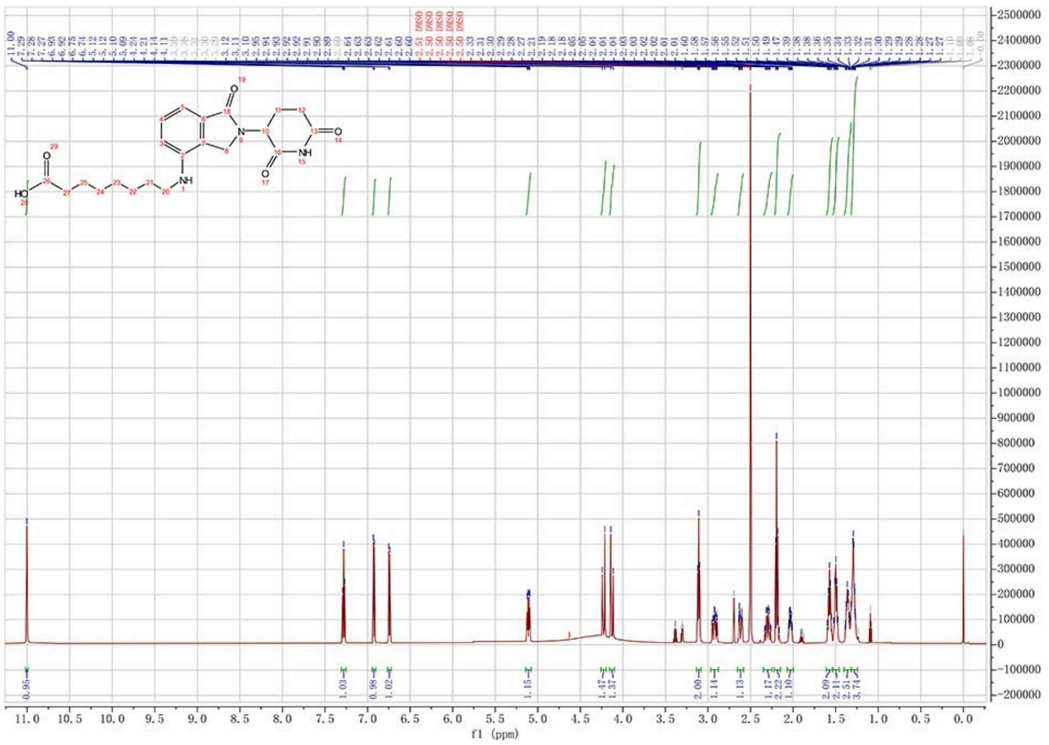

ESI-MS of compound **4b**

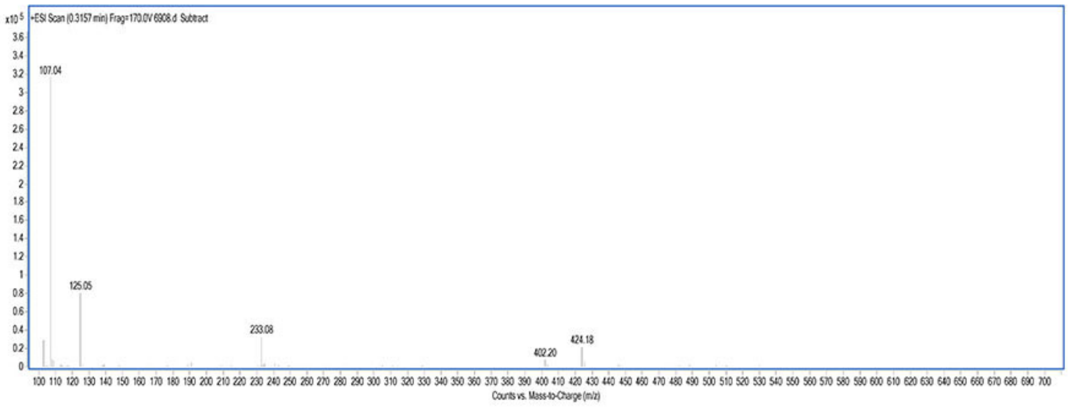

Compound 4c. Solution A: 0.1 g 4b (0.00024 mol, 1 eq) was dissolved in 3 mL DMF. Then 0.094 g HBTU (0.00024 mol, 1 eq), 0.032 g HOBt (0.00024 mol, 1 eq), and 0.156 g DIPEA (0.00134 mol, 5 eq) were added. Solution B: 0.024 g 3 - azidopropylamine (0.00024 mol, 1 eq) was mixed with TFA ($V_{3\text{-}axidopropylamine}$: VTFA = 1 : 1). Subsequent operations were consistent with the procedure for 3c. Pale yellow powder product 4c was obtained (yield 48 %).

^{1} H NMR (600 MHz, DMSO - d_6) δ 11.01 (s, 1H), 7.85 (t, J = 5.8 Hz, 1H), 7.29 (t, J = 7.7 Hz, 1H), 6.93 (d, J = 7.4 Hz, 1H), 6.74 (d, J = 8.1 Hz, 1H), 5.56 (t, J = 5.5 Hz, 1H), 5.10 (dd, J = 13.3, 5.1 Hz, 1H), 4.23 (d, J = 17.1 Hz, 1H), 4.12 (d, J = 17.1 Hz, 1H), 3.33 (t, J = 6.8 Hz, 2H), 3.17 (d, J = 5.2 Hz, 1H), 3.13 – 3.06 (m, 4H), 2.92 (ddd, J = 17.4, 13.6, 5.5 Hz, 1H), 2.63 (dt, J = 17.3, 3.8 Hz, 1H), 2.32 (qd, J = 13.2, 4.4 Hz, 1H), 2.05 (t, J = 7.4 Hz, 2H), 1.60 (dp, J = 39.8, 7.0 Hz, 4H), 1.49 (p, J = 7.4 Hz, 2H), 1.34 – 1.23 (m, 6H).

^{13}C NMR (150 MHz, DMSO - d_6): δ 173.53, 172.77, 171.88, 169.52, 144.40, 132.66, 129.83, 127.07, 112.32, 110.51, 52.10, 49.05, 46.35, 43.34, 36.35, 36.00, 31.86, 29.29, 29.28, 29.14, 29.09, 27.18, 25.86, 23.45.

ESI-MS, $[C_{24}H_{33}N_7O_4]^+$. Theoretical value: 483.26. Measured value: 484.27 & 506.25 (M + H^+ & M + Na^+).

HRMS, $[C_{24}H_{33}N_7O_4]^+$. Theoretical value: 483.2594. Measured value: 506.2485 (M + Na^+).

^{1}H NMR of compound **4c**

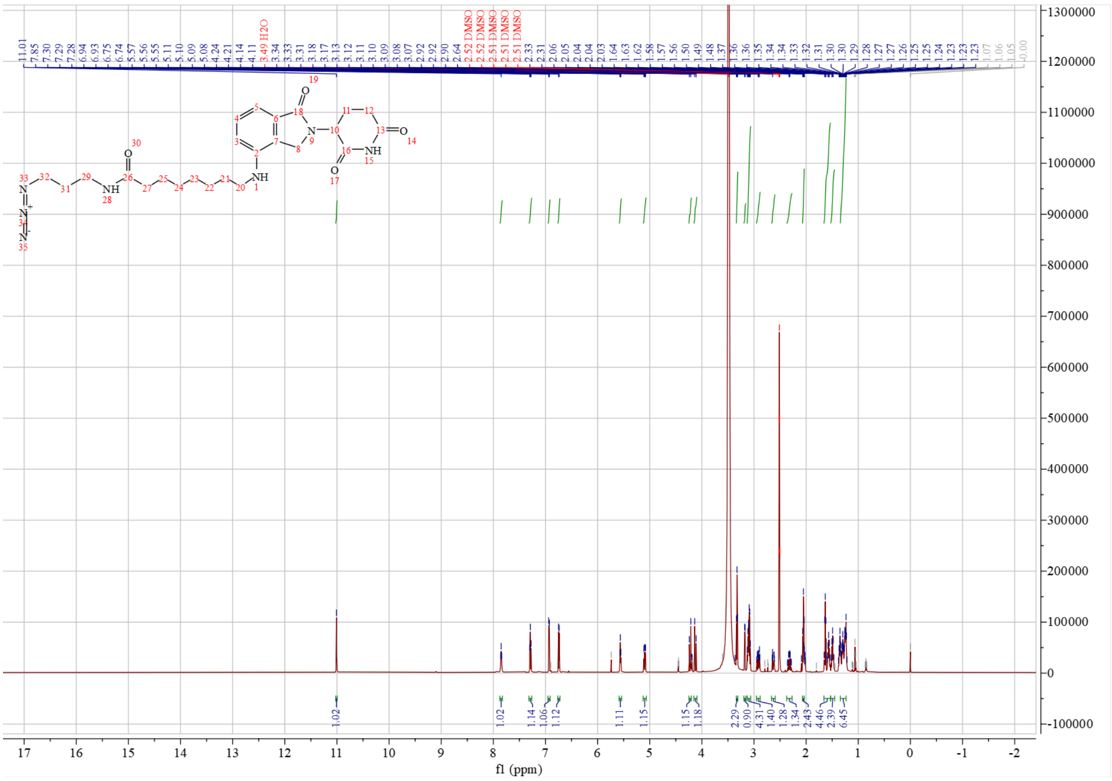

^{13}C NMR of compound 4c

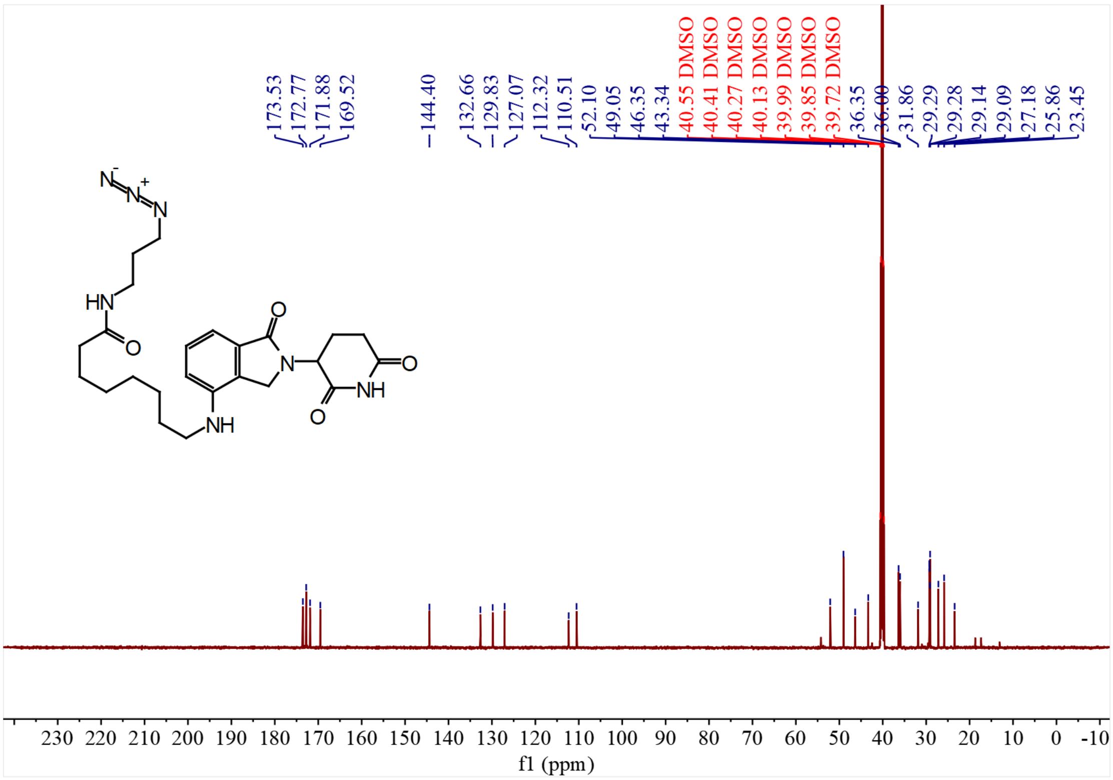

ESI-MS of compound **4c**

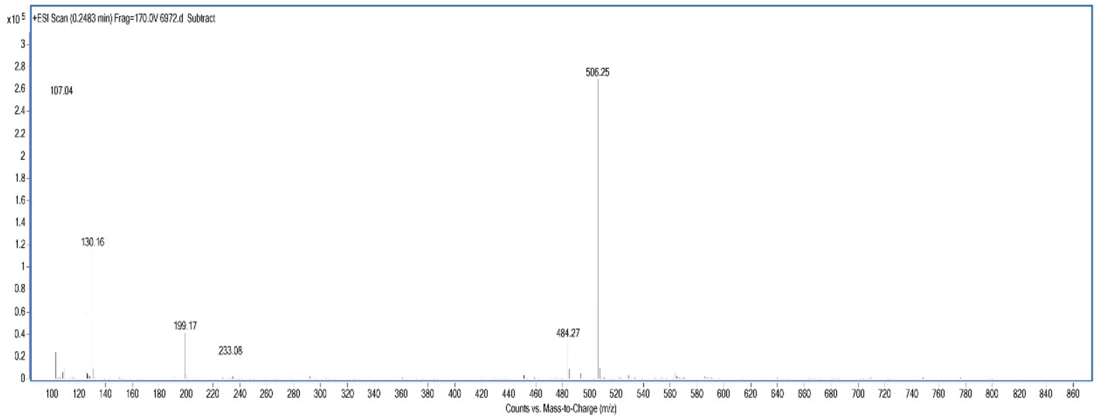

HRMS of compound **4c**

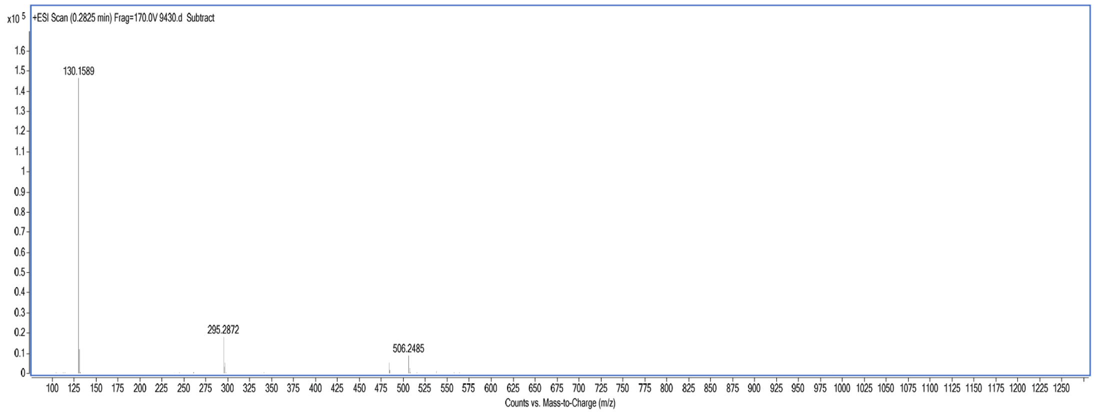

General procedure for the preparation of AS1411-PROTACs

Preparation of 2.0 mol/L TEAA buffer: First, deionized water was used to configure 2.0 mol/L glacial acetic acid solution and 2.0 mol/L triethylamine solution. After mixing the two, the pH value was adjusted to 7.0, and an appropriate amount of deionized water was added to configure it to 2.0 mol/L. 200 μL TEAA, 250 μL DMSO, 50 μL Cu (II)-TBTA complex, 50 μL sodium ascorbate (10 mmol/L) were mixed at room temperature, and then 10 μL nucleic acid sequence (0.1 OD/μL, ddH_2O, which corresponds to the 5'-terminal alkynyl-modified AS1411) and 10 μL compound (1c, 2c, 3c, 4c) (n nucleic acid sequence : n compound = 1 : 20, DMSO) were added. After mixing, they were incubated at 37 °C for 2-12 h. The target chimeric molecule was purified by HPLC, followed by vacuum centrifugal drying to evaporate the solvent. Subsequently, it was enriched using a Sep-Pak column, desalted with approximately 20 mL of sterile water, and then eluted from the desalting column with about 3 mL of 70 % methanol solution. The chimeric molecule was quantified by an ultraviolet spectrophotometer, transferred to a 1.5 mL EP tube, and dried in a centrifugal dryer. After evaporation to dryness, it was stored at a low temperature (-20 ℃). 1c: yield 61 %; 2c: yield 59 %; 3c: yield 55 %; 4c: yield 62 %.

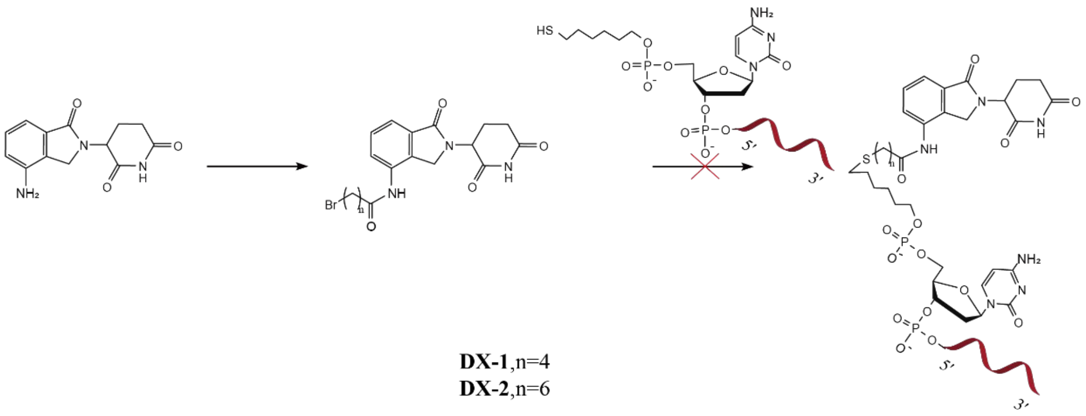

Scheme S1. A halogen-thiol-based synthetic strategy. Reagents and conditions: 5-Bromovaleric acid/7-Bromoheptanoic acid, $SOCl_2$, Anhydrous tetrahydrofuran, 80 °C, 7 h.

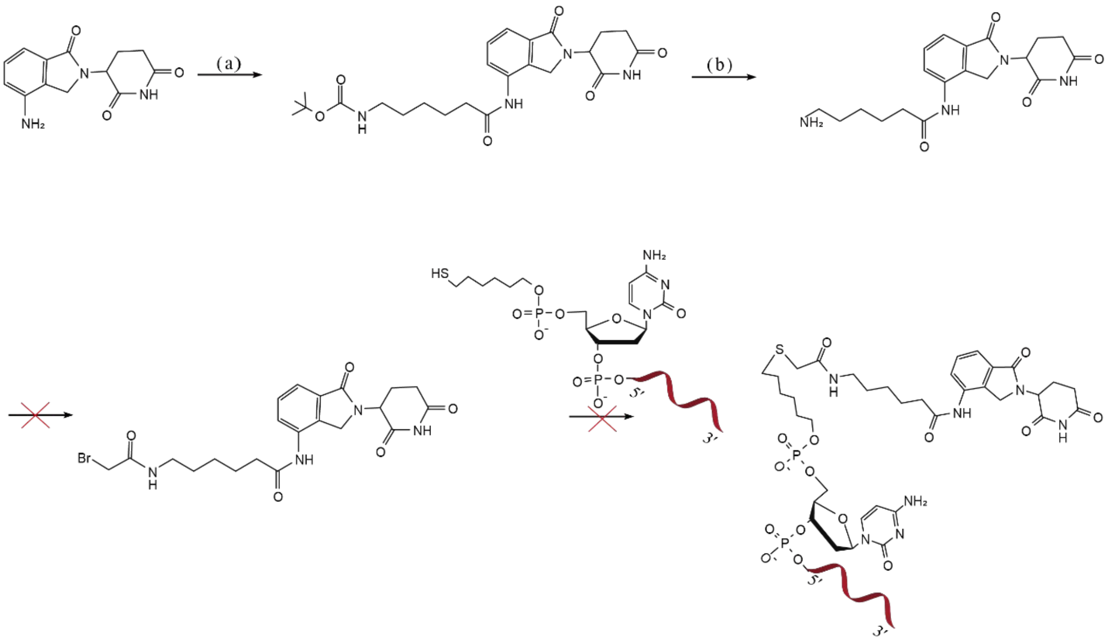

Scheme S2. A synthetic strategy based on halogenated acetyl bromide-thiol. Reagents and conditions: **(a)** 6-(boc-amino)caproic acid, pyridine, $POCl_3$, Acetonitrile, rt, 17 h; **(b)** TFA, DCM, rt, 2 h.

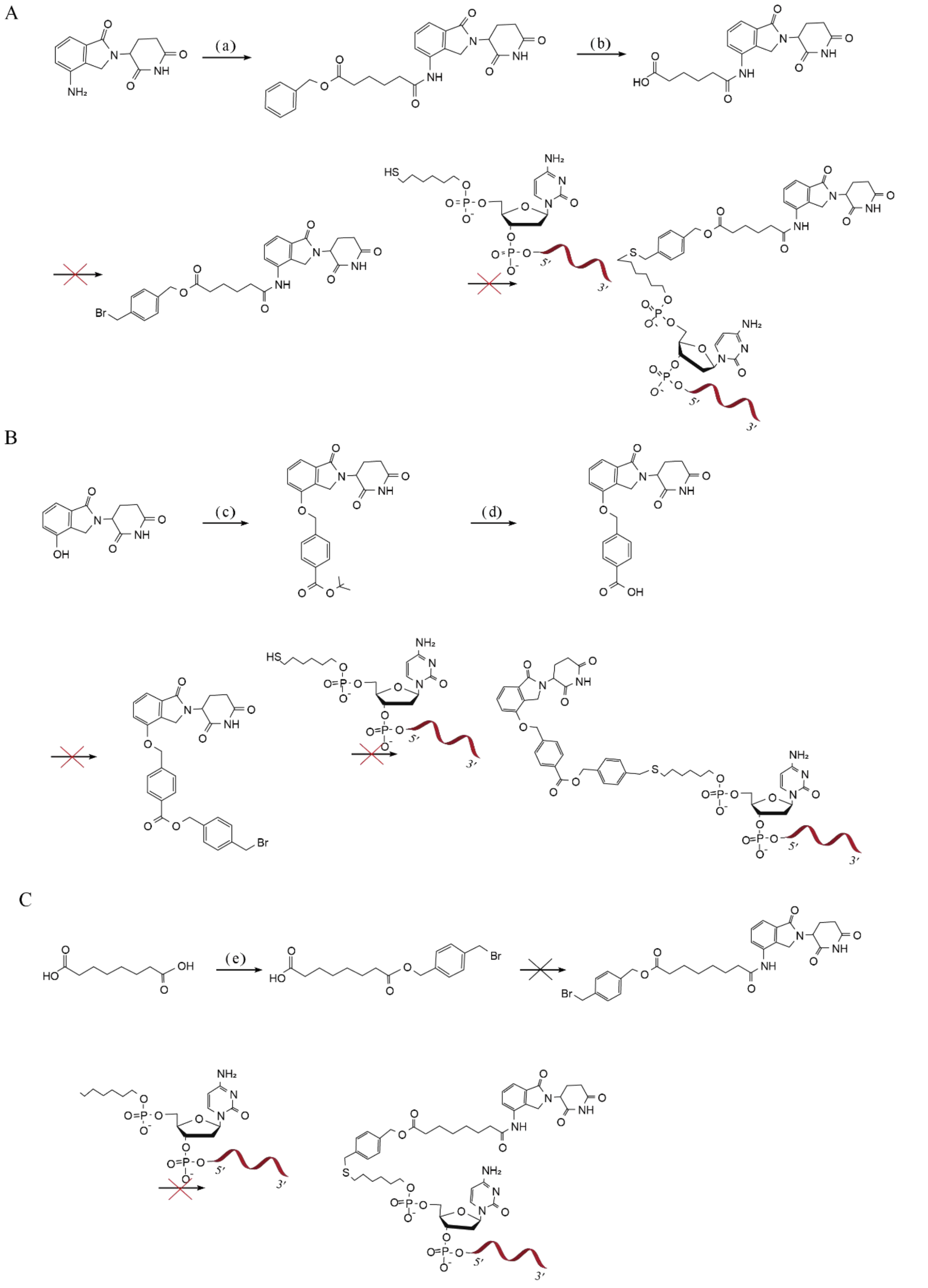

Scheme S3. A synthetic strategy based on bromobenzene-thiol. Reagents and conditions: **(a)** 6-oxo-6-phenylmethoxy-hexanoic acid, HATU, DIPEA, DMF, rt, overnight; **(b)** 10%Pd/C, MeOH, N_2, H_2, rt, 5 h; **(c)** (4-(Bromomethyl) phenyl) methanol, K_2CO_3, DMF, 50 °C, 17 h; **(d)** TFA, DCM, rt, 2 h; **(e)** (4-(Bromomethyl)phenyl) methanol, DMAP, EDAC, DCM, THF, rt, 3 h.

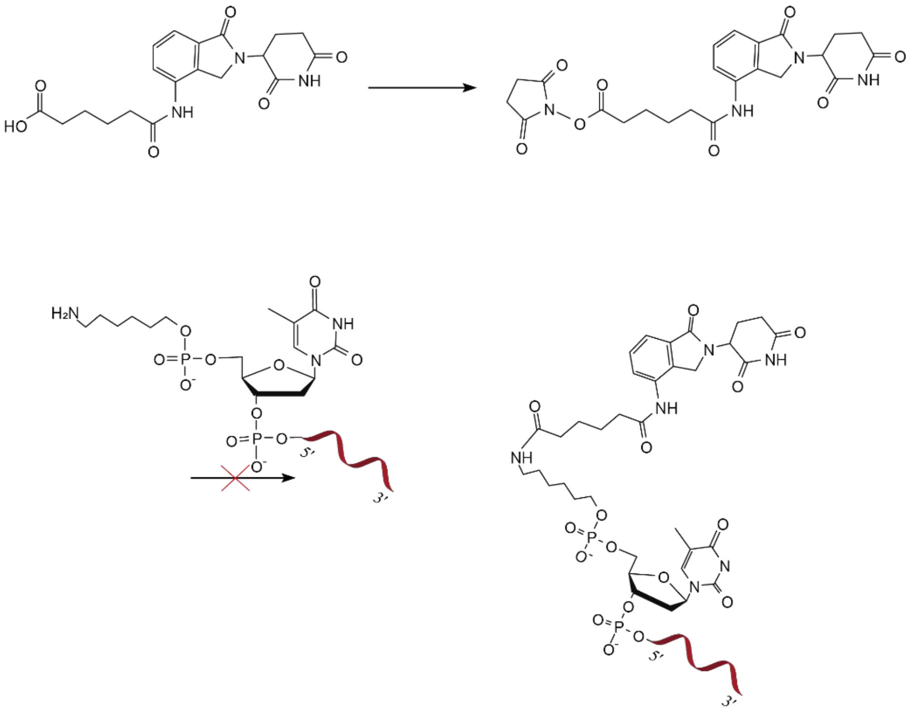

Scheme S4. A synthetic strategy based on N-hydroxy-succinimidyl esters (NHS-Esters) and amines. Reagents and conditions: NHS, EDAC, DCM, DMF, rt, overnight.

Table S1. Schematic diagram of the synthesis strategy of AS1411-lenalidomide targeting degraded chimeras.

Synthesis strategy	5′-AS1411 modification	Compound modifications	Predict product structure
Azides - alkynes	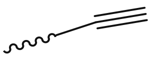	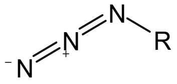	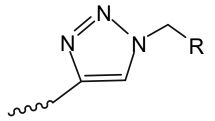
Halogens - sulfhydryl groups	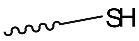	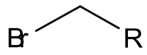	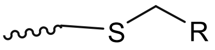
Halogenated acetyl bromide - sulfhydryl groups	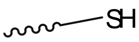	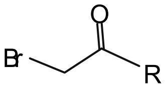	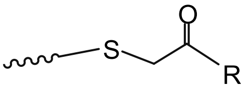
Bromine - sulfhydryl	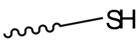	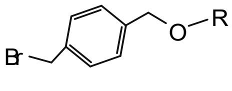	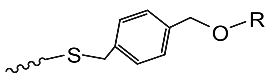
N-hydroxysuccinimide - amino	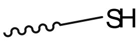	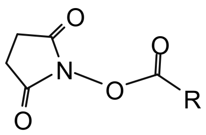	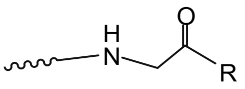

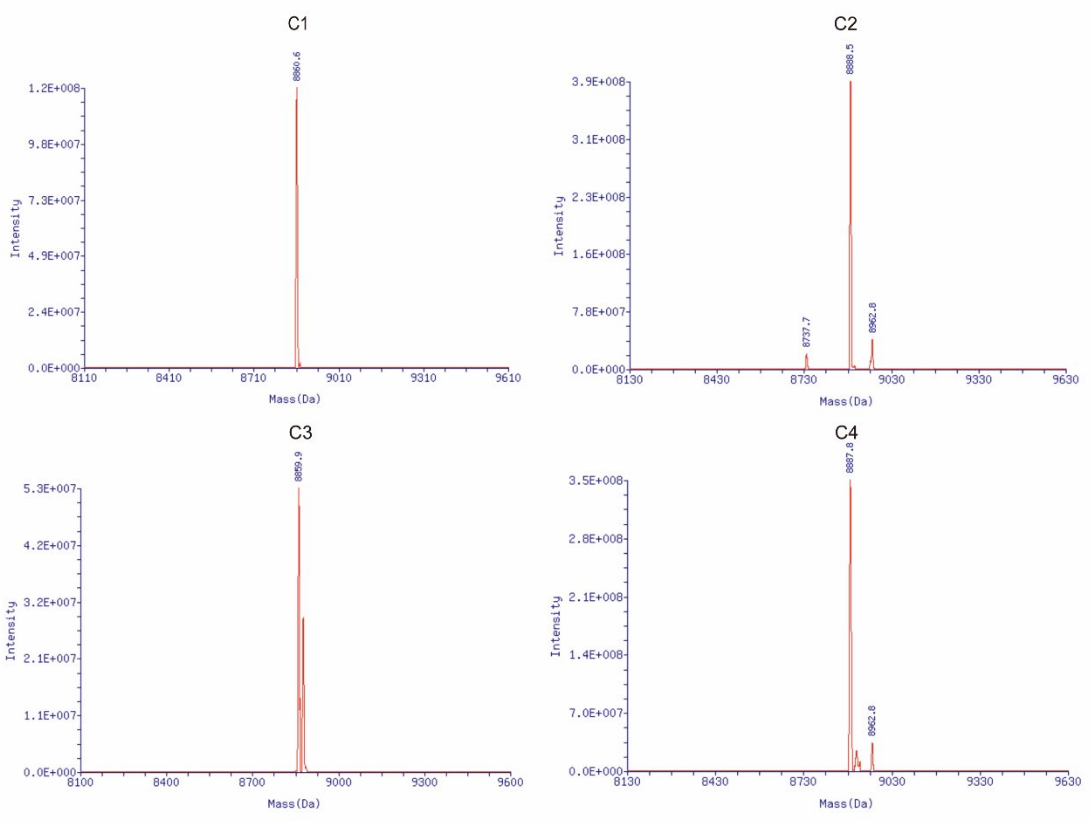

Figure S1. MOLDI-TOF-MS spectrum of C1, C2, C3 and C4.

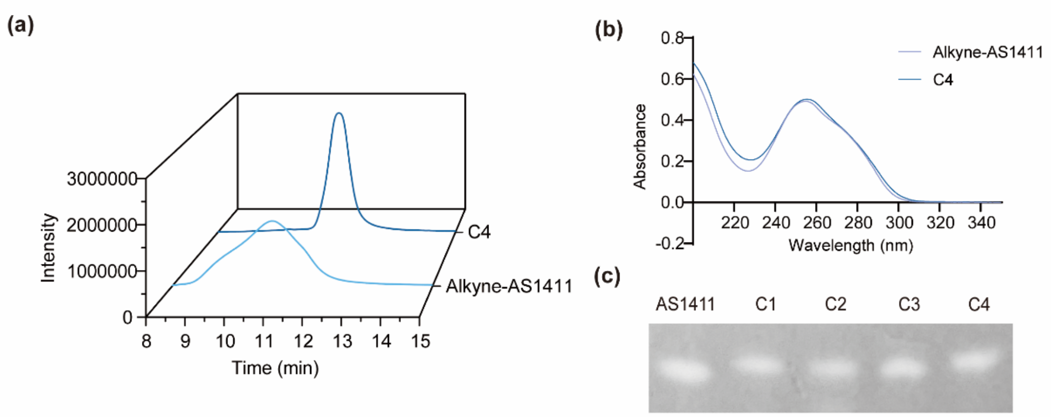

Figure S2. The characterizations of AS1411-PROTACs. **(a)** Synthesis monitoring of AS1411-PROTACs by HPLC. **(b)** The absorption spectrum of modified AS1411 and AS1411-PROTACs by UV-Vis spectrum. **(c)** Analysis of AS1411-PROTACs structure by N-PAGE electrophoresis.

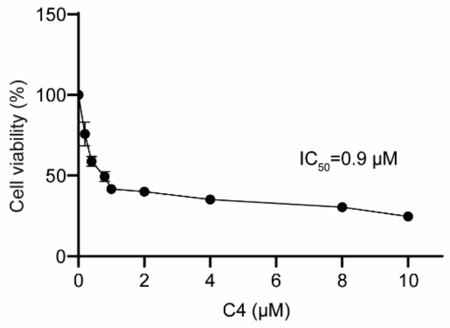

Figure S3. CCK-8 assay to detect the inhibitory effect of C4 on MCF-7 cells.

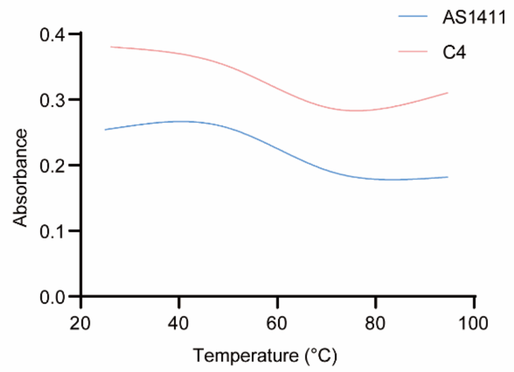

Figure S4. The sigmoidal curves responding to the Tm values of AS1411 and C4. (AS1411: Tm = 62.75 °C; C4: Tm = 66.62 °C)

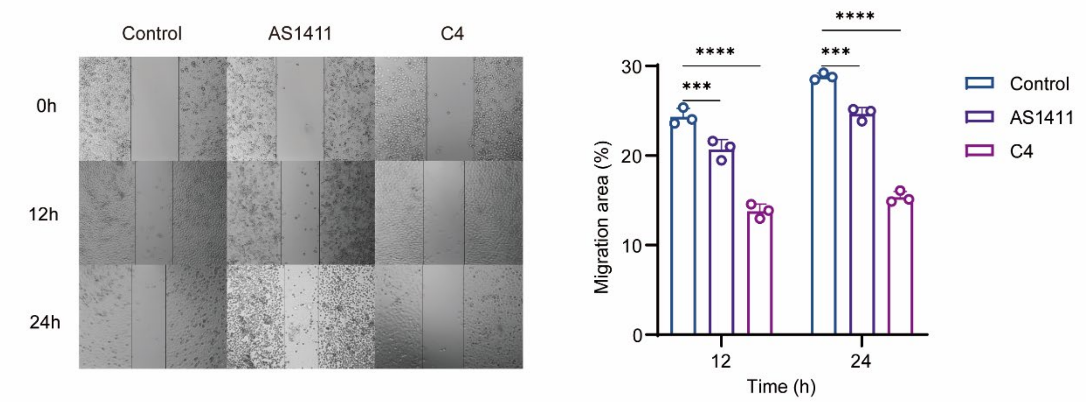

Figure S5. Cell scratch assay was used to detect the effect of AS1411 and C4 on the migration of MCF-7 cells. The colored circles (blue, purple, pink) correspond to the biological replicates of each experimental group (Control, AS1411, C4). ***P <0.001, ****P < 0.0001.

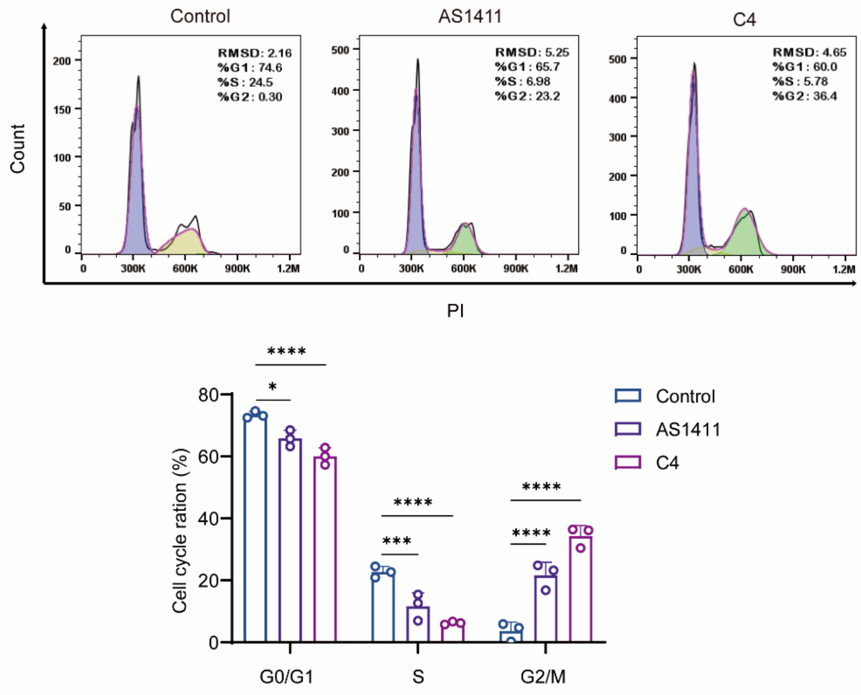

Figure S6. The cell cycle of MCF-7 cells treated with AS1411 and C4 detected by flow cytometry. The colored circles (blue, purple, pink) correspond to the biological replicates of each experimental group (Control, AS1411, C4). *P < 0.05, ***P <0.001, ****P < 0.0001.

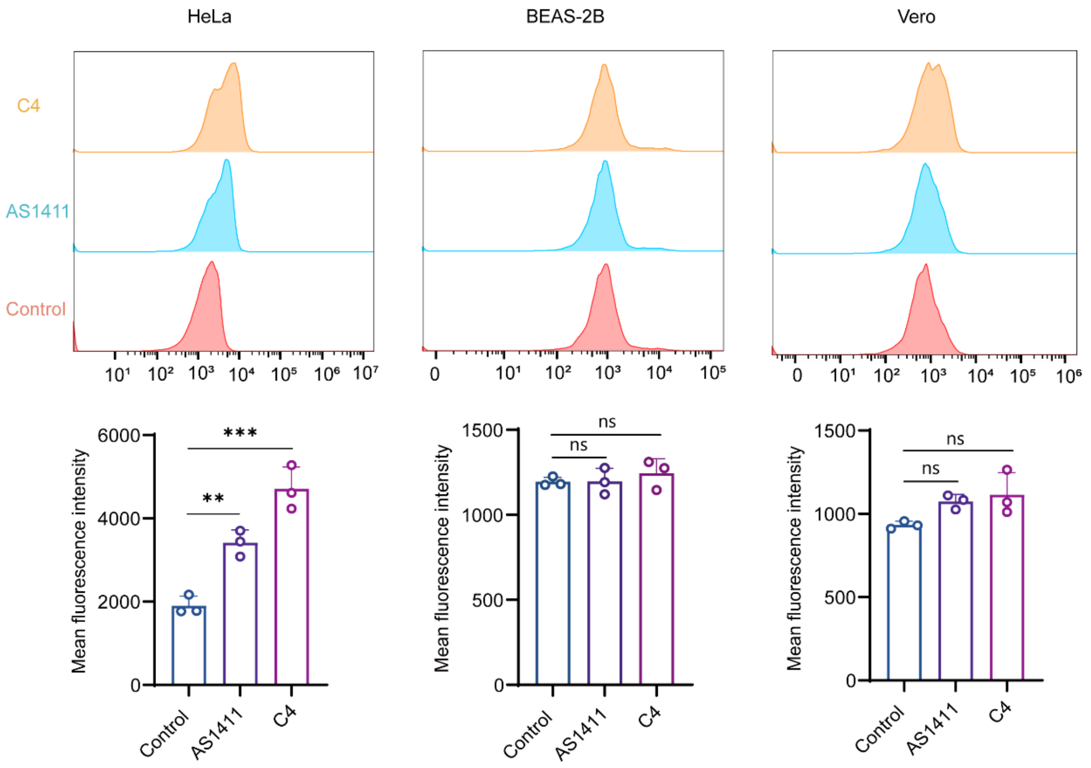

Figure S7. Representative fluorescence histograms of the AS1411 and C4 uptake pathway in HeLa, BEAS-2B and Vero cells. The colored circles (blue, purple, pink) correspond to the biological replicates of each experimental group (Control, AS1411, C4). ns: no significant difference. $^{**}P < 0.01$, $^{***}P < 0.001$.

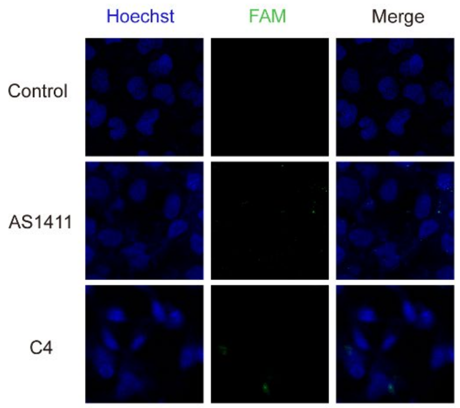

Figure S8. Confocal laser scanning microscope was used to observe the fluorescence of AS1411 and C4 uptake by BEAS-2B cells. "Hoechst" (blue) labels cell nuclei, and "FAM" (green) labels AS1411/C4.
